# Comprehensive analysis of Lon proteases in plants highlights independent gene duplication events

**DOI:** 10.1093/jxb/ery440

**Published:** 2018-12-24

**Authors:** Dikran Tsitsekian, Gerasimos Daras, Anastasios Alatzas, Dimitris Templalexis, Polydefkis Hatzopoulos, Stamatis Rigas

**Affiliations:** Department of Biotechnology, Agricultural University of Athens, Athens, Greece

**Keywords:** Expression divergence, gene duplication, gene evolution, gene paralogs, Lon protease, protein dual-targeting, protein functionalization, protein quality control, proteostasis

## Abstract

The degradation of damaged proteins is essential for cell viability. Lon is a highly conserved ATP-dependent serine-lysine protease that maintains proteostasis. We performed a comparative genome-wide analysis to determine the evolutionary history of Lon proteases. Prokaryotes and unicellular eukaryotes retained a single *Lon* copy, whereas multicellular eukaryotes acquired a peroxisomal copy, in addition to the mitochondrial gene, to sustain the evolution of higher order organ structures. Land plants developed small *Lon* gene families. Despite the *Lon2* peroxisomal paralog, *Lon* genes triplicated in the Arabidopsis lineage through sequential evolutionary events including whole-genome and tandem duplications. The retention of *Lon1*, *Lon4*, and *Lon3* triplicates relied on their differential and even contrasting expression patterns, distinct subcellular targeting mechanisms, and functional divergence. *Lon1* seems similar to the pre-duplication ancestral gene unit, whereas the duplication of *Lon3* and *Lon4* is evolutionarily recent. In the wider context of plant evolution, papaya is the only genome with a single ancestral *Lon1*-type gene. The evolutionary trend among plants is to acquire *Lon* copies with ambiguous pre-sequences for dual-targeting to mitochondria and chloroplasts, and a substrate recognition domain that deviates from the ancestral *Lon1* type. *Lon* genes constitute a paradigm of dynamic evolution contributing to understanding the functional fate of gene duplicates.

## Teaser

Despite the prokaryotic origin of ATP-dependent proteases, Lon in plant organelles acquired distinct features in terms of gene expression, protein targeting, and functionalization through independent gene duplication and evolution events.

## Introduction

Energy metabolism relies on the decomposition of polyunsaturated fatty acids and carbohydrates during storage reserve mobilization. This process is performed by intricately interconnected biochemical pathways, functional in more than one plant organelle including mitochondria, chloroplasts, and peroxisomes. While energy production is efficient, it often results in harmful by-products. In these eukaryotic organelles, the accumulation of oxidants creates an oxidative environment and causes irreversible modification of proteins (Møller *et al.*, 2007, 2011). Proteome homeostasis in plant organelles is preserved by energy-driven processive proteases that maintain protein quality control by removing defective or damaged proteins, preventing the formation of insoluble and deleterious protein aggregates.

The Lon (named from the *long* filament phenotype of bacterial mutant cells), Clp (caseinolytic protease), and FtsH (filament-forming temperature-sensitive) members of the ATP-dependent proteases of the AAA^+^ (ATPases associated with a variety of cellular activities) superfamily, evolved to degrade non-functional or short-lived regulatory proteins in plant organelles (Sakamoto, 2006; Rigas *et al.*, 2012; Janska *et al.*, 2013; Smakowska *et al.*, 2014; van Wijk, 2015). While FtsH proteases, also known as AAA proteases, are membrane integrated, Lon and Clp are soluble. Lon consists of a long N-terminal domain that possibly together with the central AAA^+^ module selectively interacts with target proteins and the C-terminal proteolytic domain with a serine-lysine catalytic dyad (Botos *et al.*, 2004; Rigas *et al.*, 2012). The AAA^+^ module regulates ATP hydrolysis and protein substrate remodeling, and is adjacent to the sensor and substrate discrimination (SSD) domain (Neuwald *et al.*, 1999; Iyer *et al.*, 2004). In both FtsH and Lon, a single polypeptide contains the ATPase and proteolytic domains, whereas in Clp these domains are kept in distinct subunits that assemble to form the proteolytic holoenzyme complex. Lon subunits of *Escherichia coli* assemble forming a ring-shaped homohexamer enclosing an internal degradation chamber accessible via an axial pore in the AAA^+^ ring, which can also interact to form a dodecamer complex (Park *et al.*, 2006; Vieux *et al.*, 2013). In contrast, the mitochondrial Lon of yeast forms a seven-membered, ring-shaped structure (Stahlberg *et al.*, 1999).

Molecular genetics in the model *Arabidopsis thaliana* has contributed to unraveling the role of Lon1 and Lon2 proteases in maintenance of organelle biogenesis and function. The *lon1* mutants exhibit growth retardation, delayed seedling establishment, aberrant mitochondrial morphology, and aberrant function of the tricarboxylic acid cycle and respiration enzymes (Rigas *et al.*, 2009*a*; Solheim *et al.*, 2012). Recently, the multiple roles of Lon1 in mitochondrial protein homeostasis, as a chaperone aiding the proper folding and stabilization of newly synthesized/imported proteins and as a protease to degrade protein aggregates, have been revealed (Li *et al.*, 2017). The *lon2* mutants show growth retardation and peroxisome-deficient phenotypes including enlargement of peroxisomes upon transition from germinating seedlings to mature plants (Lingard and Bartel, 2009). Lon2 is targeted to peroxisomes, facilitates protein import into the matrix, and controls pexophagy that is enhanced upon Lon2 deficiency (Farmer *et al.*, 2013; Bartel *et al.*, 2014; Young and Bartel, 2016). While Lon3 targeting remains unknown, it is probably expressed in sperm cells (Borges *et al.*, 2008; Rigas *et al.* 2014).

On the basis of transcriptional and translational regulation, Lon1 is dual-targeted to both mitochondria and chloroplasts by encoding two distinct protein isoforms due to twin N-terminal pre-sequences (Daras *et al.*, 2014). In contrast, Lon4 bears an ambiguous pre-sequence generating a single protein isoform with a targeting peptide recognized by the import apparatus of mitochondria and chloroplasts (Sakamoto, 2006; Ostersetzer *et al.*, 2007; Rigas *et al.*, 2014). Moreover, as predicted by molecular modeling, the architecture of the Lon1 protease core structure shows different internal geometry compared with Lon4 (Rigas *et al.*, 2014). The internal loop of Lon4 SSD shows a right-handed extension, whereas in Lon1 this domain is left-handed.

Given the progress made on the functional characterization of *Lon* genes, Arabidopsis represents a promising model system to determine gene duplication and functional divergence within the Lon proteases in plants (Koch, 2019). Our previous reports show that Lon1 and Lon4 discriminate in terms of subcellular dual-targeting mechanisms and functionalization domains (Rigas *et al.*, 2009*b*; Daras *et al.*, 2014; Rigas *et al.*, 2014). This work shows that Arabidopsis *Lon* (At*Lon*) genes present differential spatiotemporal gene expression profiles. In contrast to At*Lon1* that is widely expressed, At*Lon3* and At*Lon4* expression is exclusively restricted to male and female reproductive tissues, respectively. This mode of *Lon* paralogous gene evolution is evident only in the Arabidopsis genus emanating from the most recent whole-genome duplication (WGD) that occurred close to the base of the Brassicaceae family, also known as the α-WGD (Blanc *et al.*, 2003; Barker *et al.*, 2009), and a subsequent tandem duplication also specific to the Brassicaceae family (Haberer *et al*., 2004; Audemard *et al.*, 2012). In the wide context of land plants, comparative analysis of *Lon* phylogeny and protein structural properties provides evidence that distinct gene duplication events were the primary evolutionary force pinpointing the gradual co-evolution of alternative Lon members in embryophytes.

## Materials and methods

### Identification of Lon protease gene family members

A workflow of the genome-wide analysis to determine the evolutionary history of Lon proteases is presented in Supplementary Fig. S1 at JXB online. *Lon* protease gene information from the model plant *A. thaliana* was downloaded from The Arabidopsis Information Resources database (TAIR: http://www.arabidopsis.org/;Lamesch *et al.*, 2012). To identify *Lon* genes of unknown species, BLASTP searches were conducted using orthologous *A. thaliana* protein sequences as the query search in the publicly available phytozome database (http://www.phytozome.net/;Goodstein *et al.*, 2012). Homologs from gymnosperm species were obtained from the 1000 Plants (1KP) project (www.onekp.com;Matasci *et al.*, 2014). Homologs of animal and fungal species were obtained from the NCBI. All the data obtained are listed in Supplementary Table S1. The phylogenetic relationship of the 99 selected species was determined by the common tree taxonomy tool at NCBI (http://www.ncbi.nlm.nih.gov/Taxonomy/CommonTree/wwwcmt.cgi).

### Sequence alignment, structural modeling, and subcellular targeting prediction

Multiple sequence alignment of the identified amino acid sequences of Lon protease genes were performed by the ClustalX 1.83 software. The results were exported as GCG/MSF format and analyzed using the GeneDoc MFC Application version 2.6.0.2. The construction of all the phylogenetic trees was made by the Interactive Tree of Life (http://itol.embl.de) online platform. The alignment logos of the protein conserved domains or nucleotides were generated with WebLogo (http://weblogo.berkeley.edu/). Subcellular localization predictions of land plant Lon proteases were carried out using the TargetP 1.1 (http://www.cbs.dtu.dk/services/TargetP/), WolfPSORT (https://wolfpsort.hgc.jp/), ChloroP 1.1 (http://www.cbs.dtu.dk/services/ChloroP/), MITOPROT (https://ihg.gsf.de/ihg/mitoprot.html), and Predotar v1.04 (https://urgi.versailles.inra.fr/predotar/) servers. Structural modeling of the SSD domain of mitochondrial Lon homologs was carried out with Phyre2 using the intensive mode. Modeling was performed on the basis of known crystallographic data mainly available from AAA^+^ proteins and bacterial Lon proteases. The ribbon model was generated in PyMol (www.pymol.org). All SSDs were aligned to EcLon and then were classified according to their structure into three groups. The length and the sequences of the SSD domain of each homolog are listed in Supplementary Table S2.

### Phylogeny reconstruction

The evolutionary history was inferred by using the maximum likelihood method based on the JTT matrix-based model (Jones *et al.*, 1992). The initial tree for the heuristic search was obtained automatically by applying Neighbor–Joining and BioNJ algorithms to a matrix of pairwise distances estimated using a JTT model, and then selecting the topology with the superior log likelihood value. The analysis was performed using the MEGA 7.0 software (Kumar *et al.*, 2016).

### Synteny analysis

Synteny analyses of duplicate gene pairs and the WGD data for *A. thaliana* were achieved using the Plant Genome Duplication Database (PGDD; http://chibba.agtec.uga.edu/duplication/index/locus;Lee *et al.*, 2017).

### *In silico* expression profiles

For the expression profile analysis of *Lon* genes in Arabidopsis, ATH1 22k microarray data in the Genevestigator V3 database (Zimmermann *et al.*, 2004) and RNA sequencing (RNA-seq) data from the TravaDB database (Klepikova *et al.*, 2016) were used, and then the heat maps were constructed using the obtained gene expression data sets. Heat maps were generated using the Perseus software (version 1.5.3.2).

### *cis-*element identification and co-expression network construction

The identification of *cis*-regulatory elements was performed using the web-based analysis tool AthaMap (www.athamap.de) analyzing the promoter region extending 2000 bp upstream of the translation initiation site. The co-expression network of *Lon4* was generated by the ATTED II application (http://atted.jp/). The gene expression map was drawn by the Arabidopsis eFP browser (Winter *et al.*, 2007).

## Results

### Members of the Arabidopsis Lon family show gene expression divergence

**Fig. 1.**
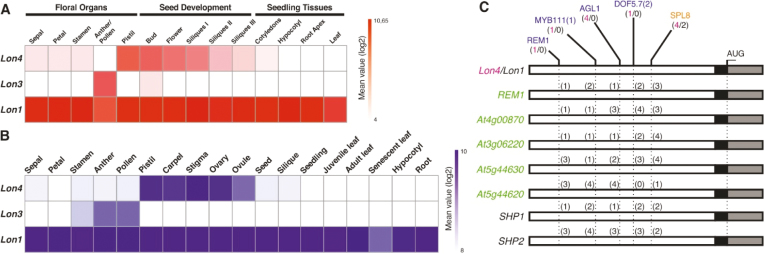
*Lon* genes in Arabidopsis show distinct expression profiles derived after gene duplication. (A and B) Heatmap plot of *Lon* gene expression obtained by (A) RNA-seq and (B) microarray data analysis. (C) The evolutionary context of CREs in *Lon4* and *Lon1* promoters contributes to their differential expression pattern in floral organs. *Lon4* contains specific CREs, depicted in blue, associated with expression restricted to pistils, which are absent from *Lon1*. Numbers in red and black depict the plethora of CREs in *Lon4* and *Lon1* promoters, respectively. Genes in green were identified by *Lon4* gene co-expression analysis (Supplementary Fig. S2A). Graphical representations of CREs do not correlate with their exact position.

Eukaryotic genomes have large numbers of duplicated genes that can evolve expression divergence, which might represent an important evolutionary mechanism for retention of duplicated genes (Haberer *et al.*, 2004; Liu *et al.*, 2011). To test whether *Lon* genes differentiate in terms of the expression profile, we analyzed data obtained by independent high-throughput approaches of the Arabidopsis transcriptome. The *Lon* genes showed reciprocal expression patterns. *Lon1* was broadly expressed across multiple organ types, whereas *Lon4* and *Lon3* appeared to have acquired a very restricted expression pattern after duplication from the common ancestor. While *Lon4* was exclusively expressed in female reproductive organs including the stigma, pistil, ovary, and ovules, *Lon3* expression was restricted to the male floral organs such as the stamen, anther, and pollen (Fig. 1A). The differential expression pattern between the *Lon* genes was also confirmed by meta-analysis of microarray data for Arabidopsis (Fig. 1B; Zimmermann *et al.*, 2004). The organ-specific expression pattern between *Lon4* and *Lon3* could only result from regulatory neofunctionalization and both genes probably represent rapidly evolving copies (Liu *et al.*, 2011). In contrast to *Lon4* and *Lon3*, *Lon1* showed a wide expression pattern, suggesting that *Lon1* retained the expression profile of the ancestral gene. Regulatory subfunctionalization is most likely to be the evolutionary fate that discriminates *Lon1* expression from that of *Lon3* and *Lon4* (Lynch and Force, 2000).

Α comparison of the genomic regulatory regions, which cover 2000 bp upstream of the translation start site, was performed to understand the molecular basis of gene expression divergence between *Lon1* and *Lon4*. The analysis led to the identification of four *cis*-regulatory elements (CREs), namely REM1, MYB111(1), AGL1, and DOF5.7(2), that exist only in the *Lon4* promoter but not in *Lon1* (Fig. 1C). However, both *Lon1* and *Lon4* share SPL8 as a common CRE, albeit SPL8 exists four times in the *Lon4* promoter and just twice in *Lon1*. The over-representation of SPL8 in the *Lon4* promoter is most probably associated with enhancement of its expression in pistils, since SPL8 has been reported to be crucial for gynoecium development and maintenance of female fertility (Xing *et al.*, 2013). This divergence in CRE constitution further supports the differential expression pattern of the two genes.

The tissue specificity of *Lon4* expression was an impetus to constitute the gene co-expression network using available public resources. The results showed a significant co-expression relationship between *Lon4* and *REPRODUCTIVE MERISTEM1* (*REM1*), *At4g00870*, *At5g44630*, *At3g06220*, and *At5g44620* sharing common CREs (Fig. 1C; Supplementary Fig. S2A). Despite the fact that *At5g44620* is missing the DOF5.7(2) element, the other four genes with a tight expression profile carried all four *Lon4*-specific elements. Remarkably, *SHP1* and *SHP2* responsible for carpel specification (Ó’Maoiléidigh *et al*., 2014), which were recently experimentally characterized as being solely expressed in pistils (Villarino *et al.*, 2016), also contained the four *Lon4*-specific CREs in their promoter regions (Fig. 1C). On the basis of their electronic fluorescent pictograph (Winter *et al.*, 2007), these genes have a similar spatiotemporal expression pattern to *Lon4*, which is exclusive in pistils (Supplementary Fig. S2B). Since certain gains and losses of CREs occur in both promoters, after gene duplication, *Lon1* and *Lon4* in addition to the dual-targeting mechanism and SSD divergence, experienced different modes of evolution of their regulatory regions that explain the differences in the expression pattern.

### Amorphic mutations caused *Lon4* and *Lon3* differentiation of targeting domains from *Lon1*

**Fig. 2.**
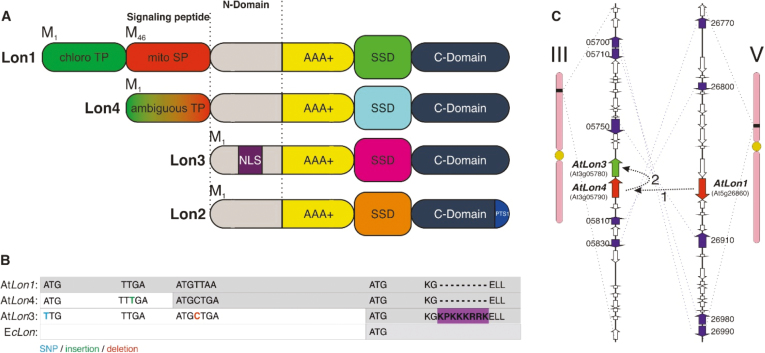
Accelerated sequence evolution after gene duplication contributed to the distinct targeting properties of Arabidopsis *Lon* genes. (A) The proteases encoded by Arabidopsis *Lon* genes acquired distinct structural domains for subcellular targeting and substrate recognition. Lon1 is dual-targeted to mitochondria and chloroplasts by twin pre-sequences, whereas Lon4 evolved an ambiguous pre-sequence at the N-terminus. Lon2 targets to peroxisomes due to a PTS1 motif at the C-terminus. The N-terminal domain of Lon3 for organellar targeting is apparently depleted and carries an NLS motif potentially for nuclear localization highlighted in purple. (B) A series of nucleotide mutations prevent the initiation of translation or abort protein synthesis upstream of the annotated ATG of *Lon3* and *Lon4*. The ORF is highlighted in gray. (C) Sequential gene duplication events and rapid evolution led to preservation of *Lon3* and *Lon4* duplicates from their common ancestor.

In addition to the differential expression pattern, *Lon* genes in Arabidopsis evolved distinct mechanisms for subcellular targeting and substrate recognition (Fig. 2A). Apart from *Lon2* that carries a PTS1 motif at the C-terminus for peroxisomal localization, it seems interesting to understand whether there is a sequence of mutation events contributing to the distinct targeting properties of *Lon1*, *Lon3*, and *Lon4*. Strikingly, *Lon3* and *Lon4* genes showed accelerated sequence evolution compared with their *Lon1* paralog. While *Lon4* has both ATG contexts similar to *Lon1* twin pre-sequences, there is a thymine (T) insertion downstream of the vicinity of the first AUG context that disrupts the ORF permitting the translation to initiate only from the second ATG (Fig. 2B; Supplementary Fig. S3A). In *Lon3*, the first ATG is mutated due to a nucleotide transition of adenine (A) to T, which restricts the initiation of translation, whereas the first nucleotide after the second ATG, that is most probably a cytosine (C), is deleted, resulting in initiation of translation from the downstream start codon (Fig. 2B; Supplementary Fig. S3A). These are all amorphic mutations, which either prevent the initiation of translation due to mutations on the initiation codon or disrupt the translation process at the region encoding the N-terminal pre-sequence permitting the translation to initiate from downstream ATG codons.

Intriguingly, the reconstitution of the mutated nucleotides reversed evolution and led to ORFs for both *Lon4* and *Lon3*. To be precise, the removal of T in *Lon4* opened the frame downstream of the first ATG and resulted in a chloroplast transit peptide (Supplementary Fig. S3B, C). In *Lon3*, the reconstitution of the first ATG and insertion of C immediately downstream of the second ATG opened a reading frame with two ATG initiation codons in-frame (Supplementary Fig. S3B). By reversion of nucleotide sequence evolution, *Lon4* and *Lon3* acquired *Lon1*-like twin pre-sequences encoding two distinct subcellular-targeted isoforms (Supplementary Fig. S3C). These—hidden by evolution—ancestral subcellular targeting properties of *Lon4* and *Lon3* revealed their common origin, suggesting that both are duplicates of *Lon1*. To support this notion further, we used the Plant Genome Duplication Database (PGDD) that provides intraplant genome alignment information to investigate evolutionary consequences of genome duplication (Lee *et al.*, 2017). The analysis revealed that *Lon4* originated from a WGD event that occurred in the herbaceous annual genus Arabidopsis and that *Lon3* is a tandem duplicate of *Lon4* as both are repeated genes located in close physical proximity to each other (Fig. 2C). Overall, the two gene copies, *Lon3* and *Lon4*, have experienced an accelerated sequence evolution in the proximity of the protein targeting region and deviated extensively from their common ancestral gene.

### Phylogenetic classification and diversification of Lon homologs

**Fig. 3.**
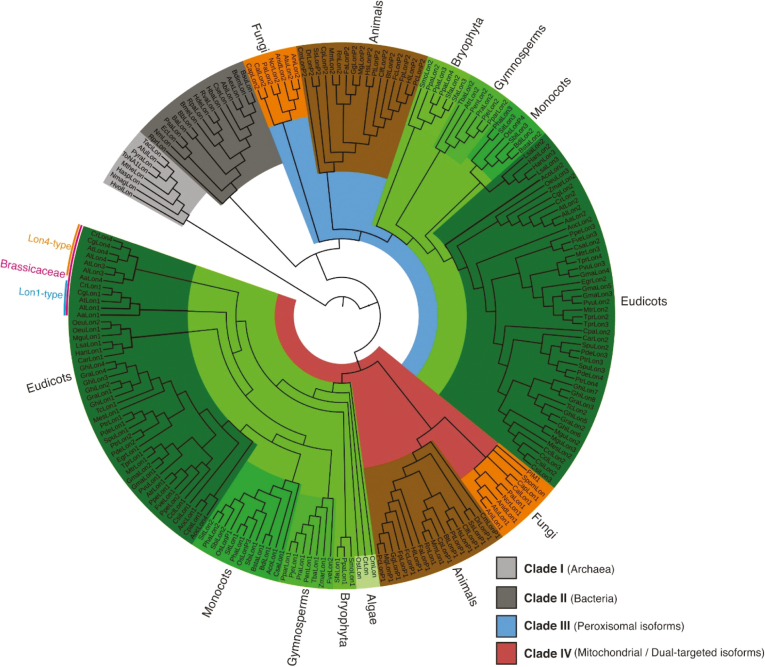
The maximum likelihood phylogeny categorized Lon homologs in four major clades. Clades I and II contain Lon of prokaryotic origin diverged in Archaea and bacteria, respectively. Clade III includes the peroxisomal Lon homologs, whereas Clade IV contains the bona fide mitochondrial Lon homologs of fungi, animals, and algae, and Lon homologs from land plants dual-targeted to mitochondria and chloroplasts.

Unlike other eukaryotic genomes, plant genomes tend to evolve at higher rates, presenting higher genome diversity and organizing genes in multicopy families (Panchy *et al.*, 2016). To explore and understand the evolutionary history of Lon proteases, we examined their phylogenetic classification in several taxa (kingdoms) including archaea, bacteria, fungi, animals, algae, and land plants. All gene entries were obtained from fully sequenced and annotated genomes (Supplementary Table S1). Maximum likelihood phylogeny reconstruction categorized the Lon homologs into four robust phylogenetic clades (Fig. 3). The *Lon* homologs of Archaea (Clade I) and Bacteria (Clade II) grouped separately from eukaryotes. Homologs of Archaea formed a monophyletic clade at the base of the phylogenetic tree due to the membrane-anchoring region emerging from the ATPase domain that discriminates the Archaea-specific LonB subfamily from the typical LonA structure of Lon homologs (Rigas *et al.*, 2012). Nonetheless, the two clades of prokaryotes were comprised of Lon homologs lacking an N-terminal targeting domain.

Based on the analysis, Lon homologs of eukaryotes with peroxisome-targeting properties comprised the third major clade (Clade III; Fig. 3). This clade was further diverged, resulting in three separate subclades of fungi, animals, and plants. This classification of Lon between the three kingdoms is indicative of the differences in the peroxisomal biochemical pathways between fungi, animals, and the green lineage. The nodes of Lon peroxisomal divergence within plants were particularly apparent with respect to their taxonomic events. Homologs from bryophyta, gymnosperms, monocots, and eudicots including Brassicaceae formed distinct subgroups. This classification of Lon peroxisomal proteases was evident by analyzing the conservation of the PTS1 motifs (Supplementary Fig. S4). The typical tripeptide serine–lysine–leucine (SKL motif) was conserved in peroxisomal Lon homologs of animal origin and mainly in higher plants including the eudicots, monocots, and conifers. The motif of bryophytic Lon homologs of lower land plants together with those of fungi deviated extensively from the typical PTS1 motif consensus. In the green lineage, there is great variability concerning the four amino acid residues upstream of the SKL motif and before the highly conserved proline (P) with respect to their taxonomic rank. From this analysis, we can suggest that plant speciation had a great impact on the diversification of the peroxisomal Lon proteases.

The clade including the non-peroxisomal-targeted Lon proteases of eukaryotes retained the largest number of homologs (Fig. 3). The phylogenetic clustering pattern divided these Lon homologs into two subclades. The first subclade included the bona fide mitochondrial-targeted homologs from algae, fungi, and animals. Concerning the biological significance of this finding, it is worth noting that algae lack a peroxisomal homolog, which is also missing from fission yeast and budding yeast (Supplementary Table S1). In contrast to hyphae-forming multicellular fungi that retained both a mitochondrial and a peroxisomal Lon isoform, yeasts, which are unicellular organisms, preserved a single mitochondrial homolog. In striking contrast, animals that like algae and fungi possess a bona fide mitochondrial Lon homolog also retained a peroxisomal copy. A possible explanation could be that animals and hyphae-forming fungi develop complex multicellular structures that require adequate energy support in contrast to unicellular organisms such as yeast or algae. Peroxisomes have an important role in lipid metabolism (Wanders *et al.*, 2010) and are implicated in ubiquitous energy metabolic pathways throughout eukaryotes, such as the β-oxidation of fatty acids (Poirier *et al.*, 2006). In filamentous fungi characterized by a multicellular architecture, the peroxisomal Lon displays a dual function as an ATP-stimulated protease and molecular chaperone to maintain proteostasis by protein quality control in the matrix (Bartoszewska *et al.*, 2012).

The phylogeny of Lons in the green lineage is reflected by their taxonomy (Fig. 3). Strikingly, the plant homologs acquired N-terminal domains for dual-targeting to mitochondria and chloroplasts (Supplementary Table S2). Like animal species, land plants evolved multicellular organs and thus they all contained peroxisomal Lon homologs (Supplementary Table S1). Taken together, we may infer that *Lon* first appeared as a single gene in prokaryotes. Then, in lower eukaryotes, *Lon* evolved to a single mitochondrial isoform and subsequently underwent duplication to generate an additional peroxisomal-targeted homolog in multicellular organisms. This event led to the emergence of *Lon* paralogs dual-targeted to mitochondria and chloroplasts in land plants.

### The majority of Lon proteases in the land plant lineage acquired an ambiguous pre-sequence

**Fig. 4.**
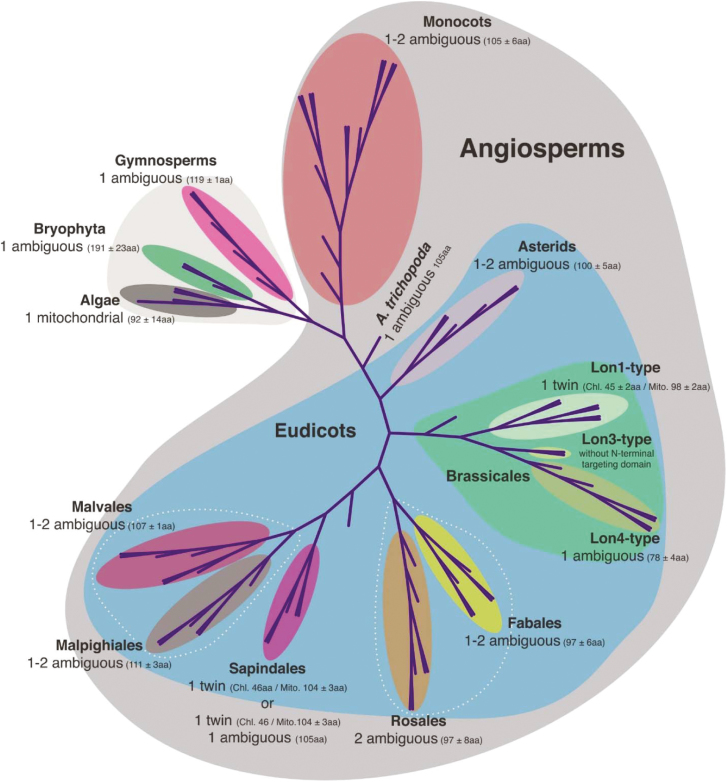
An evolutionary classification of Lon N-terminal targeting domains in plants.

To gain further insight into the evolution of the subcellular targeting properties of *Lon* homologs from land plants (Clade IV; Fig. 3), we focused on the analysis of the N-terminal targeting domains. The analysis divided algae, bryophytes, and gymnosperms from the rest of the higher plants due to the presence of only one *Lon* gene copy (Fig. 4). Nevertheless, the Lon isoform of algae was mitochondrial with a short pre-sequence, with an average polypeptide length of 92 amino acids, whereas both bryophytes and gymnosperms evolved a dual-targeted Lon homolog with an ambiguous pre-sequence with a longer polypeptide length. In the angiosperms, monocots retained up to two copies both with an ambiguous pre-sequence, which is relatively shorter than the single homolog of bryophytes and gymnosperms. While *Amborella trichopoda* is a lower angiosperm that emerged separately from the other species of monocots and eudicots, it has evolved a single *Lon* homolog with an ambiguous pre-sequence.

In eudicots, the evolution of the N-targeting domains categorized the *Lon* homologs into four groups (Fig. 4). The first group consists of four orders, namely Asterids, Fabales, Malvales, and Malpighiales, that acquired up to two Lon copies with an ambiguous pre-sequence. Rosales is a unique order which always has two Lon homologs dual-targeted to mitochondria and chloroplasts with an ambiguous pre-sequence. The orders Sapindales and Brassicales differentiated from the others due to the presence of at least one *Lon* homolog with twin pre-sequences. As a third group, the Sapindales lineage contained either a single *Lon* gene with twin pre-sequences or an additional gene copy with an ambiguous pre-sequence. The last group contains the *Lon* gene families of Brassicales with three members and distinct targeting mechanisms. While the *Lon1*-like and *Lon4*-like homologs have dual-targeting properties, they acquired different mechanisms for protein subcellular localization. The *Lon1*-like copy evolved twin pre-sequences, whereas *Lon4*-like has an ambiguous pre-sequence. In contrast, the N-terminal targeting domain was missing from the *Lon3*-like homologs. Overall, the analysis supports the notion that *Lon* homologs in land plants were formed by duplications. This is particularly evident in the Arabidopsis lineage that was derived from the Brassicaceae-specific α-WGD event leading to the formation of multigene families (Barker *et al.*, 2009).

**Fig. 5.**
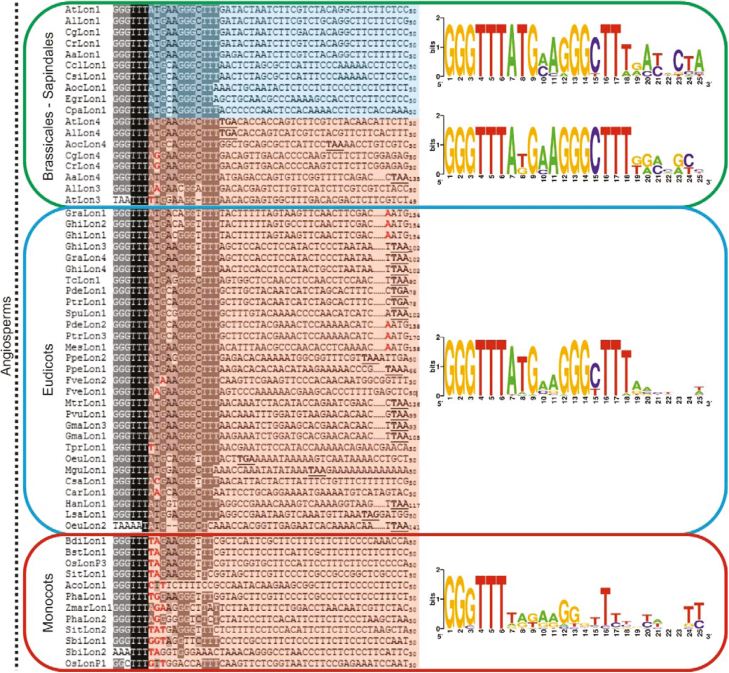
The first ATG context is highly conserved in angiosperms. In *Lon1*-like genes of Sapindales and Brassicales, the chloroplast transit peptide was retained for dual-targeting with twin pre-sequences through ORF preservation (highlighted in blue). In the *Lon* genes with an ambiguous pre-sequence for dual-targeting, the accumulation of mutational modifications led to deterioration of the chloroplast transit peptide (highlighted in red). The mutations that result in a stop codon are displayed in black underlined bold triplets, whereas the nucleotides that modify the translation initiation codon or shift the reading frame are marked in bold red.

The evolutionary history of *Lon* homologs in terms of their targeting properties was further supported by analyzing the conservation of the first ATG context in angiosperms. To identify the *Lon* homologs with twin pre-sequences, the nucleotide sequence upstream of the annotated ATG, which hereafter is called the second initiation codon, was analyzed to confirm the ORF upstream of the first ATG. This approach led to the identification of 10 out of 59 Lon homologs with twin pre-sequences (Supplementary Fig. S5), whereas the majority of the other 49 genes evolved an ambiguous pre-sequence. Multiple sequence alignment analysis revealed that in the angiosperms the first ATG was present but it was gradually deteriorated in *Lon* homologs that lost their chloroplast transit peptide upon evolution of the ambiguous pre-sequence (Fig. 5).

The highest level of conservation was evident in the orders Brassicales and Sapindales, that contain the *Lon* genes with functional twin pre-sequences due to the N-terminal extension for chloroplast targeting (Fig. 5; Supplementary Fig. S5). While the first ATG context remained highly conserved in *Lon* homologs from eudicots with an ambiguous pre-sequence, the mutational changes either modified the initiation codon or closed the reading frame only at the region encoding the N-terminal pre-sequence, resulting in initiation of translation from the downstream start codon (Fig. 5). These amorphic mutations were highly frequent in monocots.

### Lon homologs of land plants show a strong trend to acquire a Lon4-like SSD domain

**Fig. 6.**
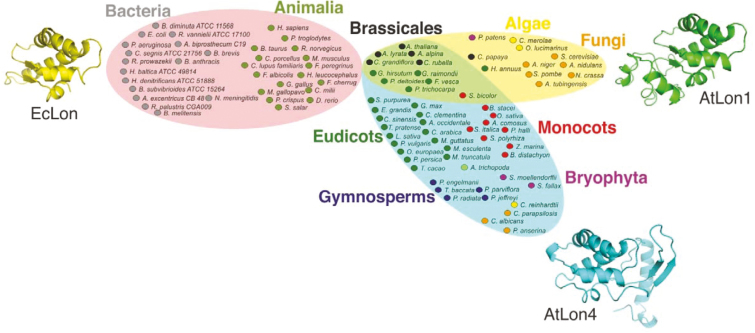
Classification of Lon homologs based on the structure of the SSD domain. Bacteria and animal SSD domains out-group from plants and fungi preserving a minimal structure. In the plant lineage, only helianthus and papaya that grouped together with most algae and fungi preserved a single gene with a Lon1-type SSD domain. Among Rosids, Brassicales in particular retained one gene copy with a Lon1-type structure and evolved copies with the Lon4-type SSD domain. The majority of land plants acquired the Lon4-type SSD domain.

The SSD domain is possibly involved in modulating selective substrate recognition by Lon proteases and thereby exhibits substantial diversity within the *Lon* homologs (Rigas *et al.*, 2009*b*). To gain a deeper insight into the evolution and functional diversification of Lon proteases, we analyzed the structure of the SSD domain (Fig. 6; Supplementary Table S3; Supplementary Fig. S6). The genome-wide study confirmed that the SSD domains of bacteria and animals are highly similar, preserving the minimal structure of *E. coli* Lon. In the green lineage, three categorization domains were formed. The first included only two species, *Helianthus annuus* and *Carica papaya*, both with a single *Lon* gene bearing an SSD domain structurally similar to At*Lon1*. Apart from these plants, this domain also included *Lon* homologs from fungi and most homologs from algae. The second domain included *Lon* homologs with more than one gene, preserving the SSD domain structure of both AtLon1 and AtLon4 from Rosids including the Brassicales and one monocot, *Sorghum bicolor*. Most land plants, namely bryophytes, gymnosperms, and angiosperms, including monocots and eudicots, were included in the third domain, which contained the *Lon* homologs with the SSD domain structure of only AtLon4. The results show a dynamic evolutionary process of Lon proteases in plants to acquire and preserve the SSD domain structure of AtLon4. The only exceptions to this rule are helianthus and papaya that preserved the SSD domain of AtLon1 and retained a single gene copy with dual-targeting properties to mitochondria and chloroplasts (Supplementary Table S2).

**Fig. 7.**
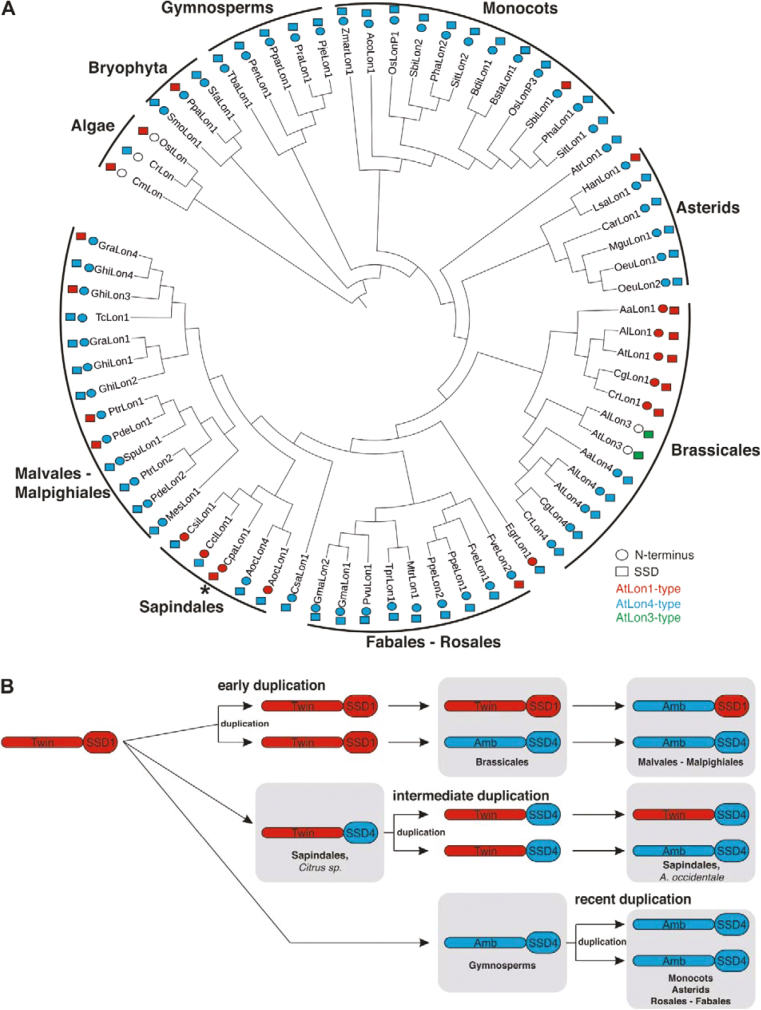
Evolutionary analysis of the structural properties and model of evolution of *Lon* paralogs in the plant kingdom. (A) Phylogenetic classification of *Lon* paralogs in the plant lineage showing the evolution of the N-terminal dual-targeting domains (squares) and the SSD domains (circles). The asterisk indicates papaya *Lon* that retained a single *Lon1*-type gene copy. (B) Model of gene duplication events driving the molecular evolution of *Lon* genes in land plants. *Lon* genes might have evolved from an ancestral gene with twin pre-sequences for dual-targeting and a Lon1-type SSD domain. Independent events of gene duplications, classified as early, intermediate, and recent, occurred together with mutational changes evolving the modern *Lon* genes in embryophytes.

Given that evolution had a great impact on the structural properties of Lon proteases in plants, we constructed the phylogeny of Lons in accordance with both domains that modulate subcellular targeting and substrate recognition (Fig. 7A). The base began with Lon homologs from algae having a mitochondrial pre-sequence and mostly preserving a Lon1-type SSD domain. The homologs from bryophytes, gymnosperms, monocots, and eudicots, mainly asterids, rosales, and fabales, almost exclusively had a typical Lon4 structure combining an ambiguous pre-sequence with a Lon4-type SSD domain.

Βrassicales, especially the Arabidopsis lineage, have evolved three genes with different subcellular targeting mechanisms and distinct SSD domain structures. Chimeric *Lon* homologs were apparent in Malpighiales, Malvales, and Sapindales, highlighting the alternation of the Lon1-type and Lon4-type structural features. This analysis yielded four major findings. First, the Arabidopsis lineage—probably due to a specific sequence of genome duplication events—evolved three *Lon* paralogs. Secondly, papaya, though it belongs in Brassicales, remained the only species with a single Lon1-type gene, providing evidence that unlike Arabidopsis taxa, papaya missed critical duplication events. Thirdly, in most land plants excluding algae, the Lon4 type is dominant. Finally, the chimeric *Lon* genes demonstrated rapid evolution to combine both Lon1-type and Lon4-type features.

## Discussion

### Arabidopsis Lon duplicates acquired diverged targeting mechanisms, asymmetric sequence evolution, and gene expression divergence

Gene duplication has contributed to the evolution of novel structures or agronomic traits, which are thought to be important in plant adaptation to variable abiotic and biotic environments, in speciation and diversification (Soltis *et al.*, 2009; Panchy *et al.*, 2016). Polyploidy, or WGD, is the most extreme gene duplication mechanism that results in a vast increase in genome size and gene sets. Apart from WGD, subgenomic mechanisms also contribute to gene duplications (Zhang, 2003). Tandem (or local) duplication is attributed to unequal crossing-over events and leads to a cluster of at least two paralogous sequences with no or a few intervening gene sequences. While WGD leads to a dramatic increase of the gene content, it is a rare phenomenon. In contrast, tandem duplication, although affecting a limited number of genes, can modify the gene number upon every meiotic division. Both WGDs and tandem duplications account for the majority of gene duplicates in plants. However, the predominant fate of most duplicates is either to become a non-functional pseudogene or gene loss through the diploidization process that ultimately leads to the return of many genes to single copy (gene fractionation). Genome-wide studies have revealed that the preservation of duplicated genes depends on nucleotide sequence modifications resulting in a differential expression pattern, distinct subcellular localization properties, and functional divergence (Ganko *et al.*, 2007; Carretero-Paulet and Fares, 2012; Byun and Singh, 2013; Jiang *et al.*, 2013).

Interestingly, *Lon* genes of Arabidopsis were preserved as paralogs after duplication because they fulfill these criteria. In terms of expression pattern, *Lon1* is expressed in all organs, whereas *Lon4* expression is mainly detected in pistils, buds, and at the first stages of silique development, suggestive of a highly specific function. The differences in expression pattern can be explained by the presence or absence of certain portions of the regulatory regions. After duplication, *Lon4* experienced evolutionary changes in the regulatory region, gaining specific CREs associated with gene expression specificity in pistils. In particular, CREs such as REM1, MYB111, AGL1, and DOF5.7 in the promoter region of *Lon4* seem to be the key to understanding the mechanisms that underlie the expression differences between *Lon1* and *Lon4*. These elements are *Lon4* specific, as they do not exist in the *Lon1* promoter, and presumably play an important role in shaping the expression pattern of *Lon4*. Furthermore, these regulatory sites are shared by the promoters of three genes including *REM1* that are co-expressed with *Lon4*. They are also present in the promoters of *SHATTERPROOF1* (*SHP1*) and *SHATTERPROOF2* (*SHP2*) identified by a genome-wide transcriptional analysis of genes involved in the development of pistils (Villarino *et al.*, 2016). The fact that the four CREs are highly conserved among these genes that show specific expression in pistils further implies a minimal context of CREs necessary to drive gene expression specificity. In contrast to *Lon4*, the expression of *Lon3* was restricted to the male reproductive parts of the flower. On the basis of these findings, the pre-duplication expression pattern is likely to be a broad expression pattern similar to that of *Lon1*. Furthermore, it is widely appreciated that as younger duplicates, *Lon4* and *Lon3* evolved rapidly, acquiring a more divergent and restricted expression pattern compared with *Lon1* (Casneuf *et al.*, 2006; Ganko *et al.*, 2007; Liu *et al.*, 2011). Hence, *Lon3* and *Lon4* gene duplicates were preserved by dividing the ancestral expression pattern of *Lon1* between the duplicates, whereas *Lon3* and *Lon4* differentiated by each gaining a discrete expression pattern.

The distinct subcellular targeting properties of Arabidopsis *Lon* genes increased the retention of duplicates. Both *Lon4* and *Lon3* gene copies showed accelerated sequence evolution compared with their *Lon1* paralog. A series of amorphic mutations gradually modified the first ATG context, inhibiting the initiation of translation or disrupting the reading frame. Consequently, *Lon4* acquired an ambiguous pre-sequence for dual-targeting to chloroplasts and mitochondria, whereas *Lon1* preserved both ATGs in-frame. The mutational changes of *Lon3* were more severe in the vicinity of both ATGs, probably resulting in the loss of a functional N-terminal targeting domain.

Homology modeling revealed that structurally the Arabidopsis Lon proteases deviate from the hexameric bacterial and human Lon proteases, fitting best with the heptameric yeast structure (Rigas *et al.*, 2014). This is most probably attributed to the primary amino acid sequences of Arabidopsis Lon sequences, which are significantly longer than the bacterial and human Lon proteases (Rigas *et al.*, 2009*b*; Venkatesh *et al.*, 2012). Moreover, the internal geometry is different between *At*Lon1 and *At*Lon4. The internal loop domain of *At*Lon4 shows a right-handed extension, whereas in *At*Lon1 it is left-handed. The Arabidopsis *Lon* genes showed an asymmetric rate of amino acid sequence evolution at the highly variable SSD domain (Rigas *et al.* 2009*b*; Daras *et al.*, 2014). The accumulation of neomorphic mutations led to non-synonymous amino acid substitutions contributing to the modification of the core domain likely to be involved in substrate recognition. Taking these points into consideration, it appears reasonable to suggest that evolution of *Lon* genes in Arabidopsis is an ongoing process contributing to better fitness, selection, and adaptation of gene duplicates. *Lon1* has probably retained the ancestral form, whereas *Lon3* and *Lon4* have evolved independently.

### *Lon* genes were formed by duplication events during the evolution of land plants

The phylogenetic analysis of *Lon* genes provided evidence of evolutionary divergence among species. The prokaryotes together with unicellular eukaryotic species, namely yeasts and algae, retained a single gene copy, despite the divergence of subcellular targeting and substrate recognition domains. Unlike these eukaryotic organisms, hyphae-forming fungi, animal species, and land plants acquired an additional peroxisomal copy. While animals strictly preserved one mitochondrial and one peroxisomal gene copy, the land plants evolved small *Lon* gene families with members acquiring dual-targeting properties to chloroplasts and mitochondria and differentiated functional domains. It is worth mentioning that bacteria and animals preserved a minimal structure of the SSD domain, whereas plants and fungi evolved an extended domain. These results coincide with the concept that plants, as sessile organisms, are constantly exposed to acute environmental conditions and thus, through adaptive evolution, preserved the duplicated *Lon* genes to maintain proteostasis in organelles. Furthermore, gene expression of Arabidopsis *Lon* duplicates specifically in floral organs supports the view of Lon proteases having evolved to satisfy the growing demands for energy and carbon during sexual reproduction.

Our analysis also revealed that the driving force towards the evolution of Lon proteases in land plants was a series of independent gene duplication events together with the accumulation of asymmetric mutations in the duplicates. Even though the exact phylogenetic placement of these events is an issue that cannot be easily resolved, we propose a model that discriminates *Lon* evolutionary fate in three phases, hereafter called early, intermediate, and recent gene duplication events (Fig. 7B). Early duplication in Arabidopsis led to the differentiation of gene duplicates. Consistent with previous reports (Liu *et al.*, 2014), we provide evidence that *Lon4* was generated by a WGD event and *Lon3* by a tandem duplication that placed it next to *Lon4*. The broad expression pattern, the subcellular targeting mechanism by twin pre-sequences, and the divergence within functional domains, reveal that the pre-duplication ancestral gene unit is likely to be similar to *Lon1*.

These observations agree with the widely appreciated concept that the Arabidopsis lineage has experienced three WGD events (Blanc *et al.*, 2003; Bowers *et al.*, 2003; Barker *et al.*, 2009). The most recent event is called the *At*-α (alpha), the intermediate event is referred to as the *At*-β (beta), and the oldest is the *At*-γ (gamma). While *At*-γ is shared with other Rosids, including papaya (Carica), poplar (Populus), and grape (Vitis), the most recent WGD, *At*-α, is specific to the Brassicaceae family (Schranz and Mitchell-Olds, 2006) and accounts for ~2500 pairs of duplicated genes in the Arabidopsis genome (Blanc *et al.*, 2003; Bowers *et al.*, 2003). Even though *Carica papaya* belongs together with Arabidopsis in the same order, Brassicales, its genome did not undergo the two most recent WGD events, *At*-α and *At*-β (Barker *et al.*, 2009). Surprisingly, the ancestral genome of papaya is the only one among the land plants containing a single-copy *Lon* gene that shares outstanding similarities with Arabidopsis *Lon1* with respect to the twin pre-sequences and the structure of the SSD domain. These observations suggest that among the order Brassicales, papaya carries the pre-duplication ancestral *Lon* gene, which is placed at the beginning of the model for *Lon* evolution in plants (Fig. 7B). Moreover, the duplication of *Lon4* is evolutionarily recent because it was derived from the Arabidopsis-specific *At*-α WGD after divergence from the Brasssica lineage.

Nevertheless, papaya seems to be the sole exception to the rule of *Lon* evolutionary history. The analysis revealed that the majority of plant species evolved genes that are similar to the Lon4 type (Fig. 7B). These duplicates acquired an ambiguous pre-sequence to drive the protein isoform to both chloroplasts and mitochondria and an SSD domain that deviates from the Lon1 structure. Although this gene form dominates, *Citrus* sp. retained an intermediate form, which preserved the twin pre-sequences but lost the Lon1-type structure of the SSD domain.

In conclusion, our results provide evidence of the complex dynamics underlying the evolution of Lon proteases by gene duplication and mutational events. Despite the complexity, this genome-wide study sheds light on the ancestral *Lon* gene form in plants and pinpoints the trend of evolutionary history to enhance plant adaptability to harsh environmental conditions, counteracting the organellar oxidative environment and irreversible modification of proteins. Overall, the results provide valuable information for further studies on the roles of Lon proteases in the protein quality control of plant organelles.

## Supplementary data

Supplementary data are available at *JXB* online.

Fig. S1. Schematic presentation of the workflow applied to determine the evolutionary history of Lon proteases.

Fig. S2. *Lon4* is co-expressed with pistil-specific genes.

Fig. S3. Correction of *Lon3* and *Lon4* mutations reconstitutes the N-terminal targeting domain to *Lon1* twin pre-sequences.

Fig. S4. Conservation and dissimilarities of the PTS1 motif in *Lon* peroxisomal homologs.

Fig. S5. The N-terminal extension of *Lon1*-like homologs from Sapindales and Brassicales encoding a chloroplast transit peptide for dual-targeting with twin pre-sequences.

Fig. S6. Molecular modeling of the sensor and substrate discrimination (SSD) domain reveals the functional features of Lon proteases.

Table S1. List of *Lon* genes used in the study and their accessions.

Table S2. Dual-targeting properties and sensor and substrate discrimination (SSD) domain structure of *Lon* homologs from land plants.

Table S3. Protein length and the co-ordinates of the SSD domain of the bacterial and eukaryotic Lon homologs targeted to mitochondria (fungi and animals) or dual-targeted to chloroplasts and mitochondria (plants).

Supplementary Figures S1-S6

Supplementary Table S1

Supplementary Table S2

Supplementary Table S3
