# Supplementary material for: Comprehensive analysis of Lon proteases in plants highlights independent gene duplication events
Source: J Exp Bot. 2018 Dec 24;70(7):2185–97. doi: 10.1093/jxb/ery440 (PMC6460959; doi:10.1093/jxb/ery440)
Supplement: Supplementary Figures S1-S6 [file ery440_suppl_supplementary_figures_s1-s6.pdf]

**Supplementary data**

# Comprehensive analysis of Lon proteases in plants highlights independent gene duplication events

Dikran Tsitsekian, Gerasimos Daras, Anastasios Alatzas, Dimitris Templalexis, Polydefkis Hatzopoulos and Stamatis Rigas

This PDF includes: Figs S1-S6

**Supplementary Fig. S1.**

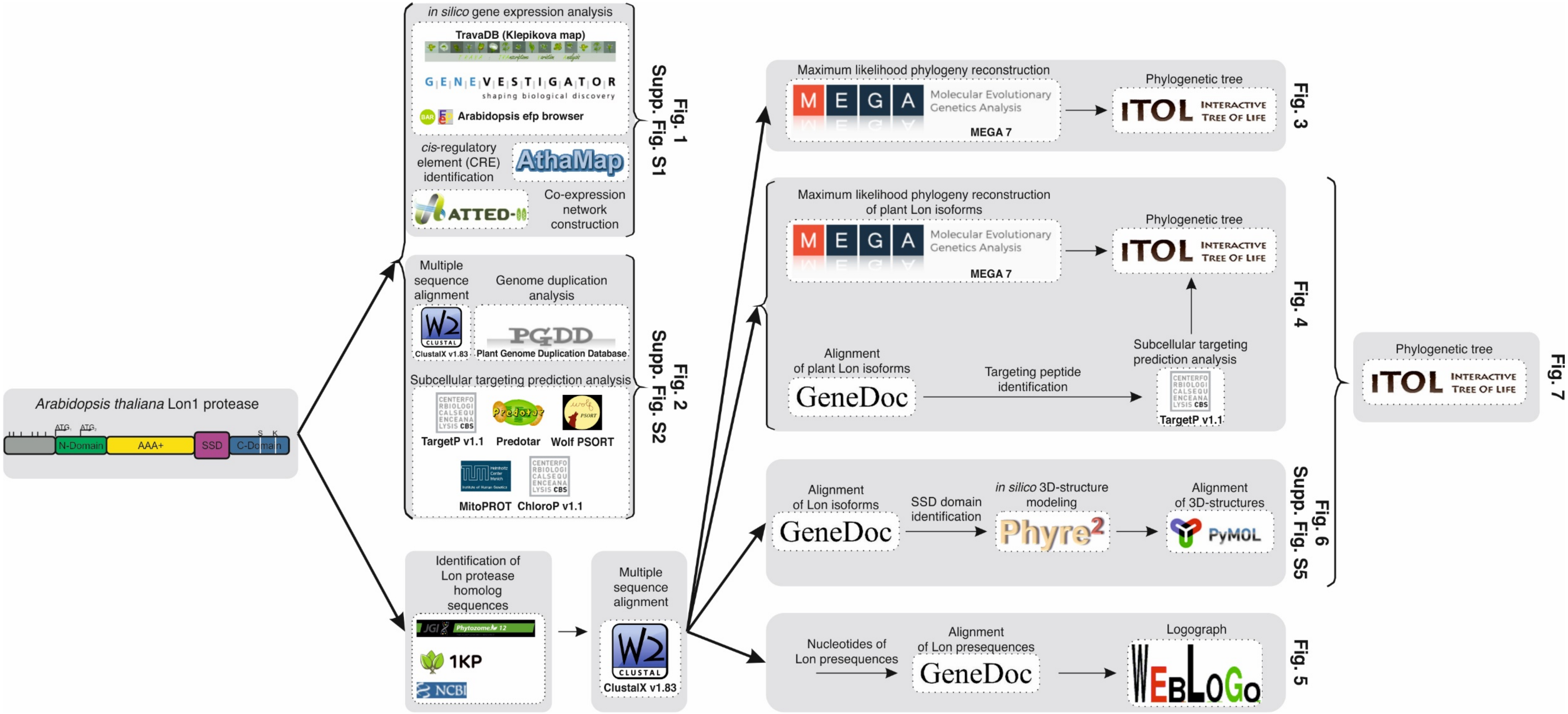


**Supplementary Fig. S1.** Schematic presentation of the workflow applied to determine the evolutionary history of Lon proteases.

**Supplementary Fig. S2.**

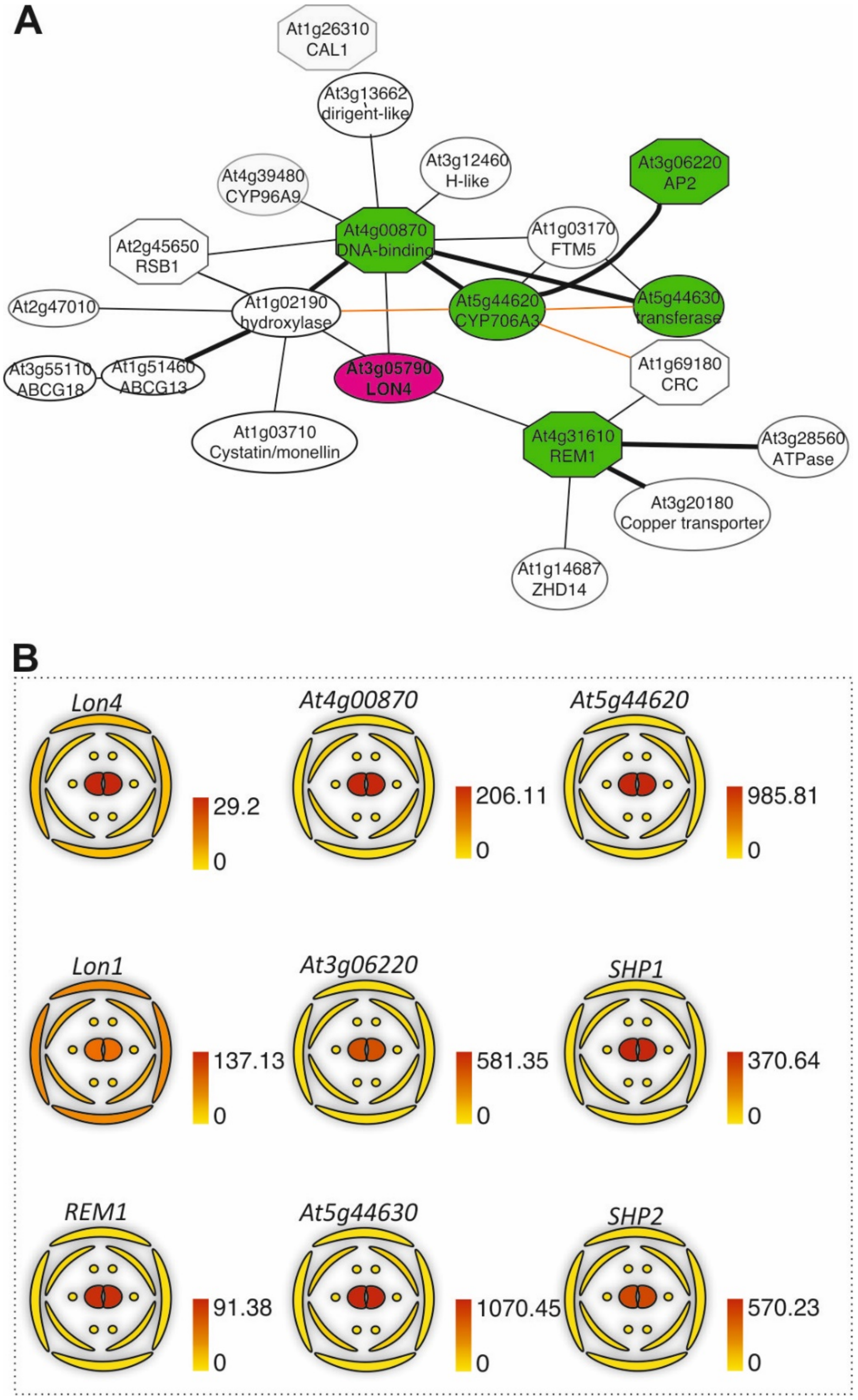


**Supplementary Fig. S2.** *Lon4* is co-expressed with pistil specific genes.

(A) Co-expression analysis of genes that share the *Lon4*-specific CREs. The octagons represent transcription factors. Conservation of co-expression profile identified in non-plant species is shown by the orange line.

(B) Graphical display of genes expression pattern within the floral organs. Levels of absolute expression are shown by the heat-map.

**Supplementary Fig. S3.**

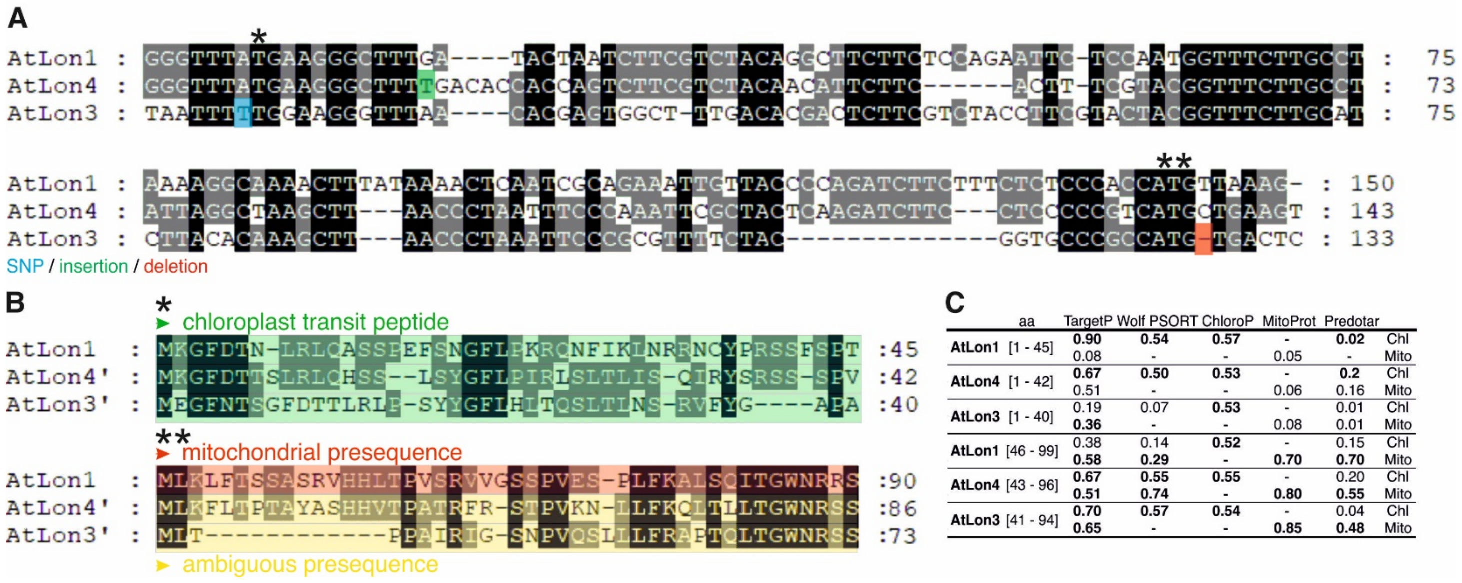


| aa | TargetP | Wolf PSORT | ChloroP | MitoProt | Predotar | |
|---|---|---|---|---|---|---|
| **AtLon1** [1 - 45] | **0.90** | **0.54** | **0.57** | - | **0.02** | Chl |
| | 0.08 | - | - | 0.05 | - | Mito |
| **AtLon4** [1 - 42] | **0.67** | **0.50** | **0.53** | - | **0.2** | Chl |
| | 0.51 | - | - | 0.06 | 0.16 | Mito |
| **AtLon3** [1 - 40] | 0.19 | 0.07 | **0.53** | - | 0.01 | Chl |
| | **0.36** | - | - | 0.08 | 0.01 | Mito |
| **AtLon1** [46 - 99] | 0.38 | 0.14 | **0.52** | - | 0.15 | Chl |
| | **0.58** | **0.29** | - | **0.70** | **0.70** | Mito |
| **AtLon4** [43 - 96] | **0.67** | **0.55** | **0.55** | - | 0.20 | Chl |
| | **0.51** | **0.74** | - | **0.80** | **0.55** | Mito |
| **AtLon3** [41 - 94] | **0.70** | **0.57** | **0.54** | - | 0.04 | Chl |
| | **0.65** | - | - | **0.85** | **0.48** | Mito |

**Supplementary Fig. S3.** Correction of *Lon3* and *Lon4* mutations reconstitutes the N-terminal targeting domain to *Lon1* twin presequences.

(A) Nucleotide sequence alignment of the N-terminal domain showing the two in-frame ATG of *Lon1* and the mutations present at this region of *Lon4* and *Lon3*. Single and double asterisks show the position of the first and second translation initiation codon.

(B) Reversion of the amorphic mutations reconstitutes *Lon4* and *Lon3* N-terminal targeting domains to twin presequences.

(C) Computational predicted subcellular targeting of Lon4 and Lon3 acquired twin presequences. The first N-terminal extension of Lon4 and Lon3 encompassing the amino acid residues between the two ATG initiation codons, is predicted to be similar to a transit peptide. The N-terminal portion downstream of the second ATG possesses properties for dual-targeting. The values range from 0 (no prediction score) to 1 (highest score probability).

**Supplementary Fig. S4.**

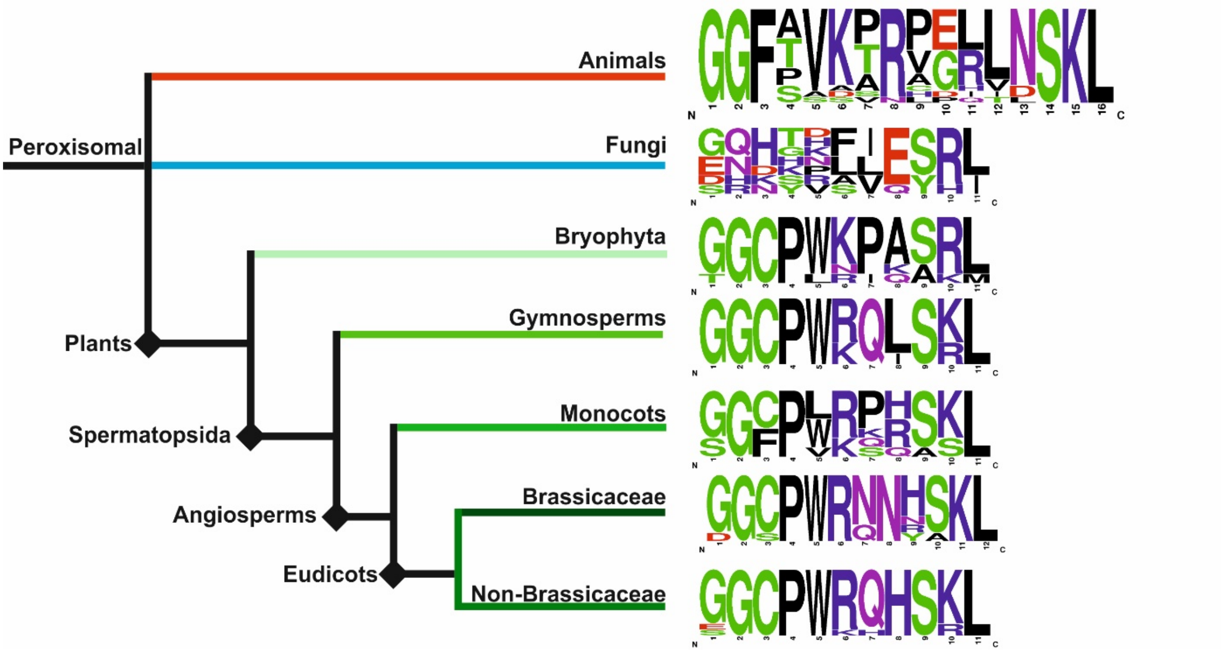


**Supplementary Fig. S4.** Conservation and dissimilarities of the PTS1 motif in *Lon* peroxisomal homologs.

**Supplementary Fig. S5.**

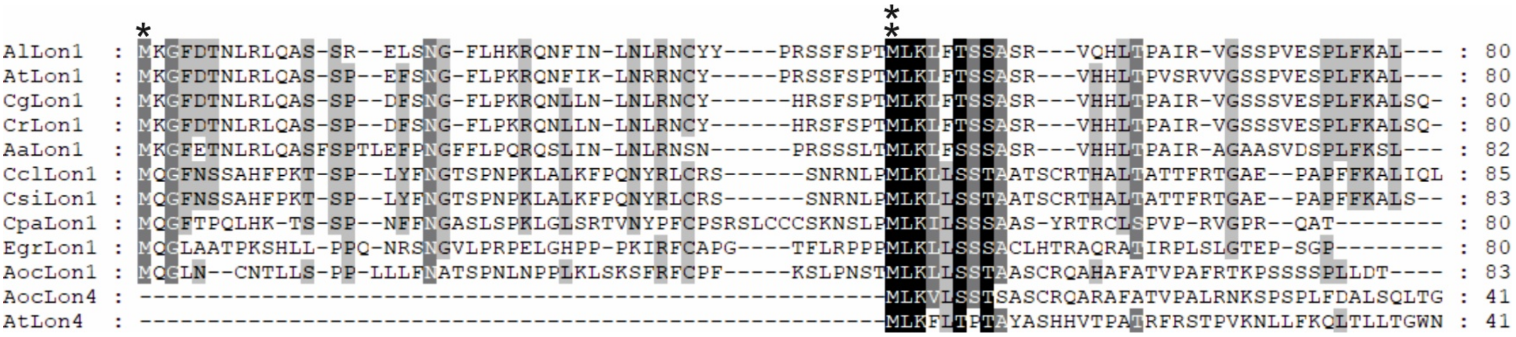


**Supplementary Fig. S5.** The N-terminal extension of *Lon1*-like homologs from Sapindales and Brassicales encoding a chloroplast transit peptide for dual-targeting with twin presequences. Single and double asterisks show the position of the first and second translation initiation codon.

**Supplementary Fig. S6.**

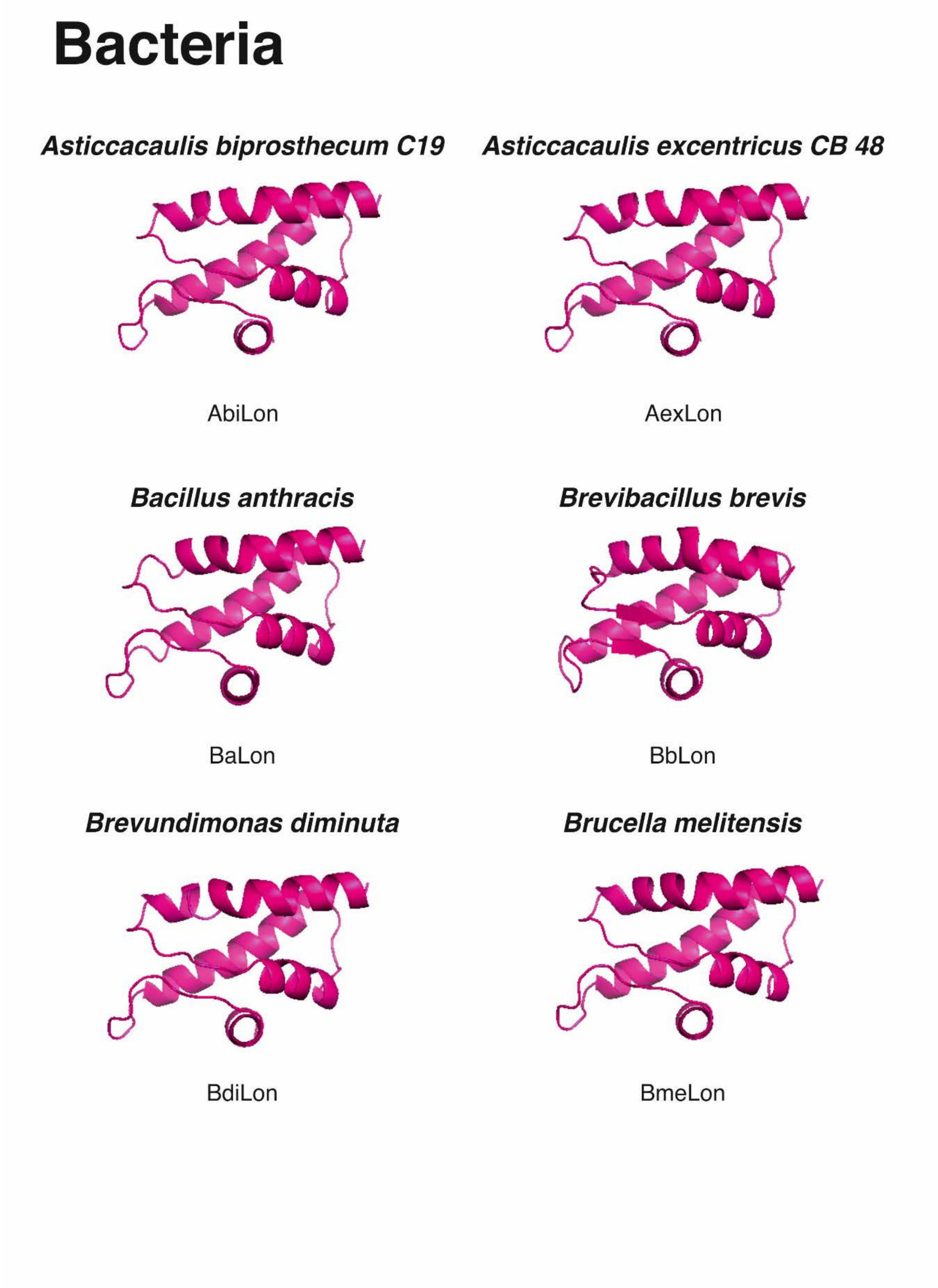

# Bacteria

***Brevundimonas subvibrioides***

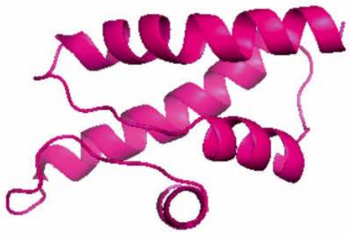

BsuLon

***Caulobacter segnis***

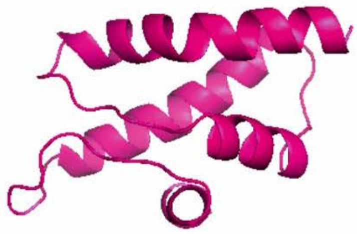

CseLon

***Escherichia coli***

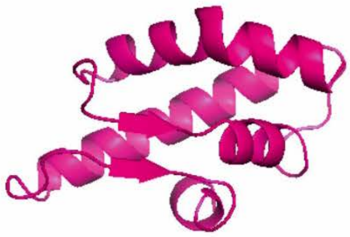

EcLon

***Hirschia baltica***

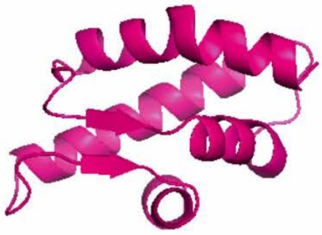

HbaLon

***Hyphomicrobium denitrificans***

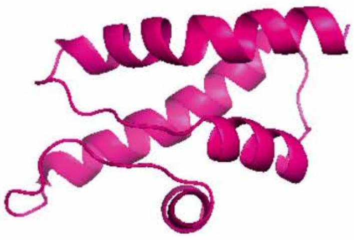

HdeLon

***Neisseria meningitidis***

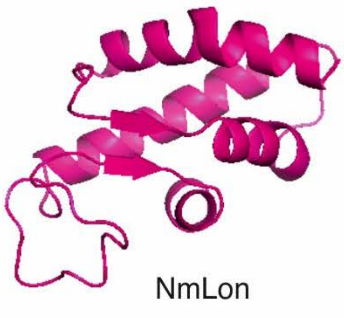

NmLon

# Bacteria

***Pseudomonas aeruginosa***

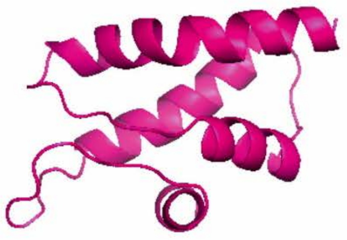

PsaLon

***Rhodopseudomonas palustris CGA009***

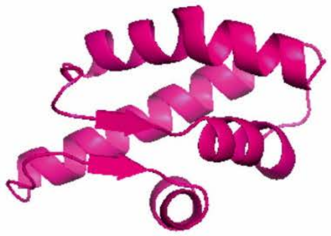

RpaLon

***Rickettsia prowazekii***

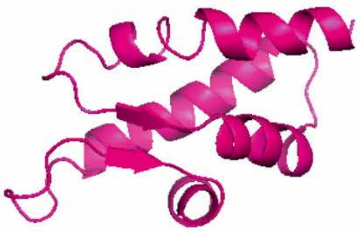

RprLon

***Rhodomicrobium vannielii***

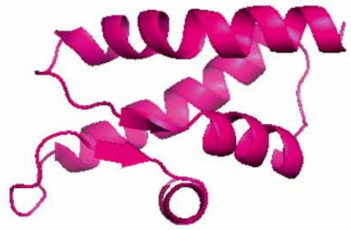

RvaLon

# Fungi

***Aspergillus nidulans***

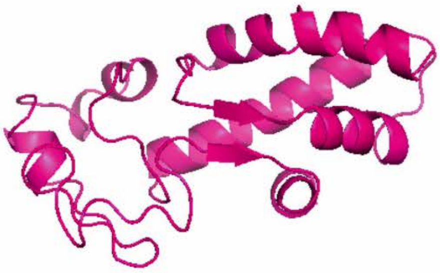

AnidLon1

***Aspergillus niger***

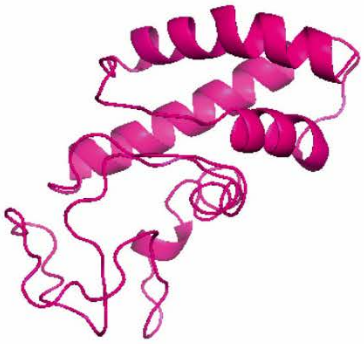

AniLon1

***Aspergillus tubingensis***

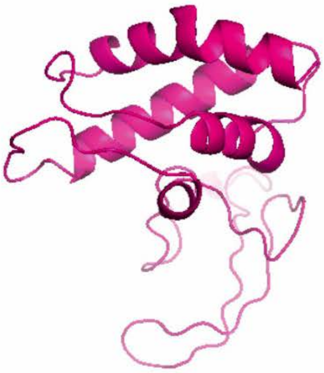

AtuLon1

***Saccharomyces cerevisiae***

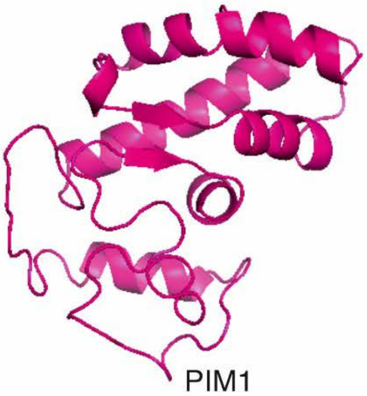

PIM1

***Schizosaccharomyces pombe***

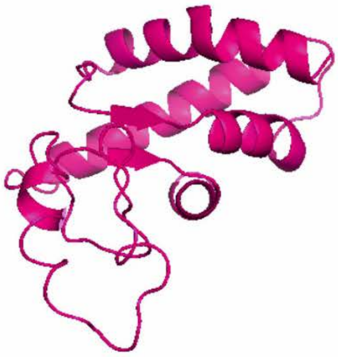

SpomLon

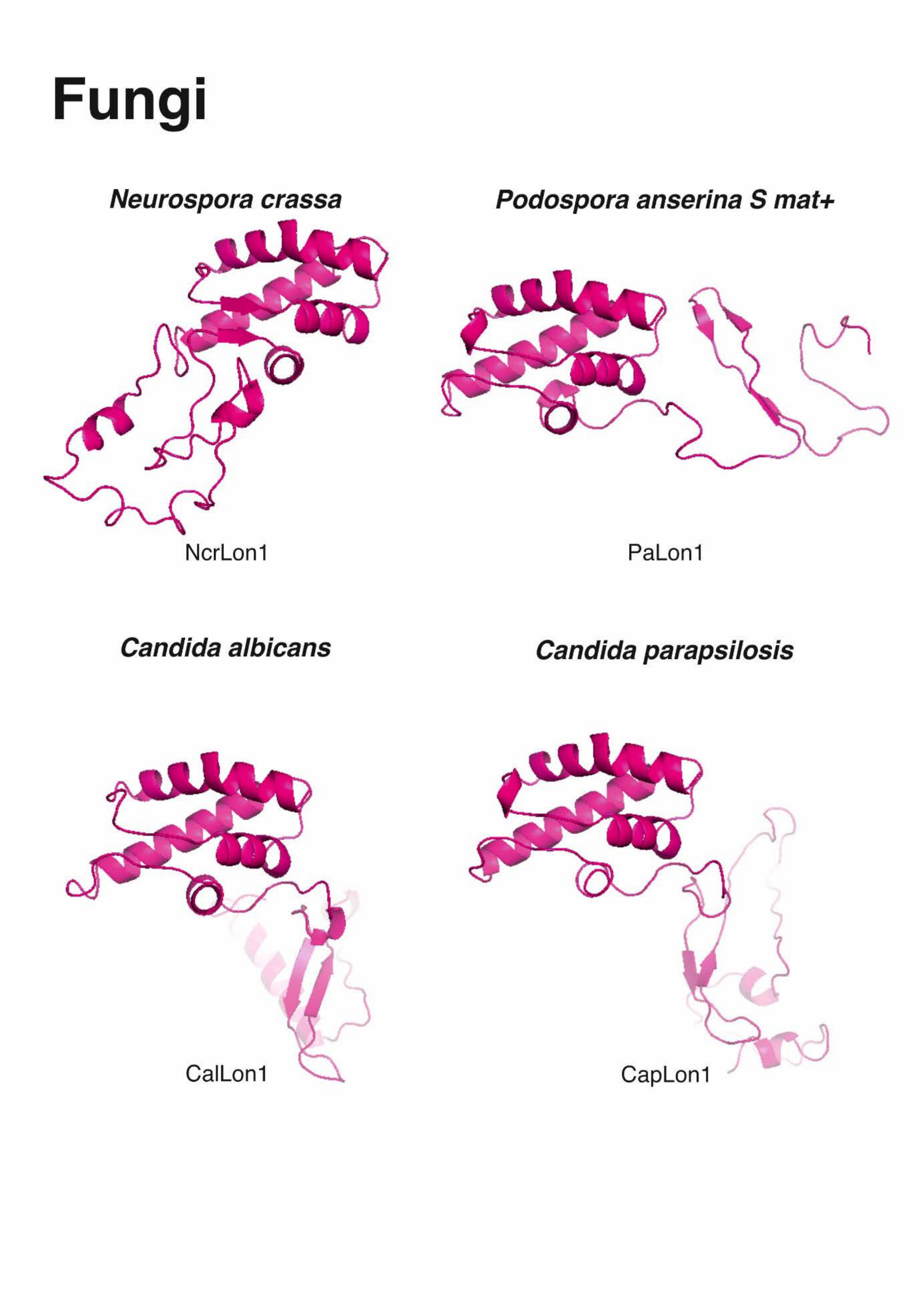
Fungi
Neurospora crassa
NcrLon1
Podospora anserina S mat+
PaLon1
Candida albicans
CalLon1
Candida parapsilosis
CapLon1

# Algae

***Chlamydomonas reinhardtii***

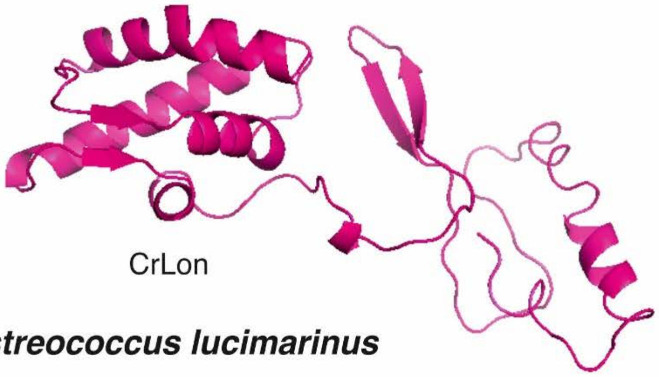

CrLon

***Ostreococcus lucimarinus***

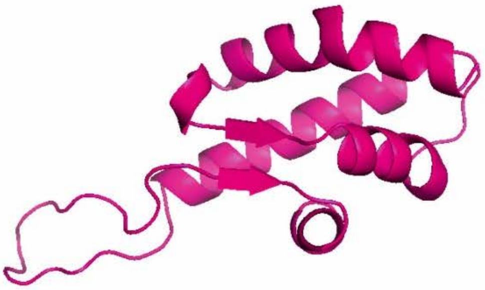

OstLon

***Cyanidioschyzon merolae***

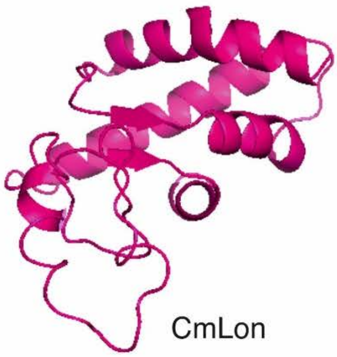

CmLon

# Bryophyta

***Physcomitrella patens***

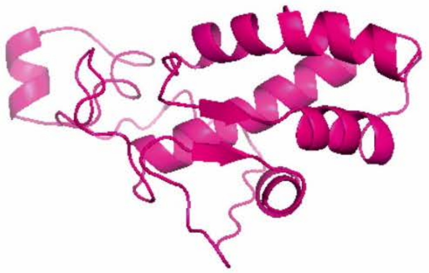

PpaLon1

***Sphagnum fallax***

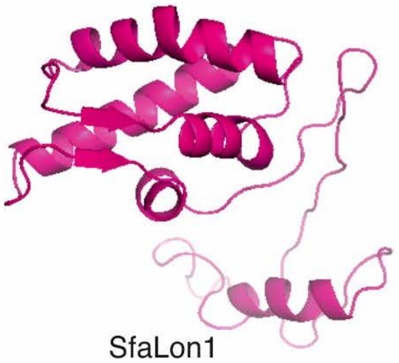

SfaLon1

***Selaginella moellendorffii***

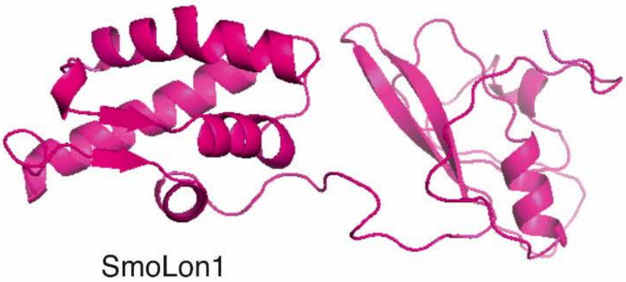

SmoLon1

# Gymnosperms

***Picea engelmannii***

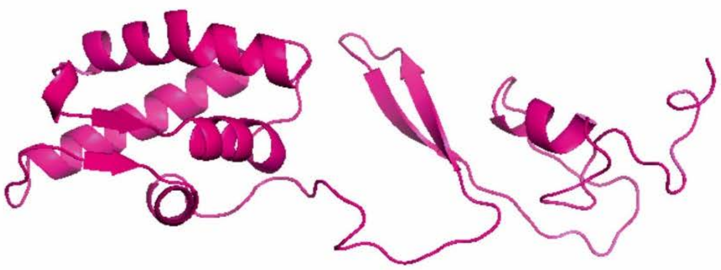

PenLon1

***Pinus jeffreyi***

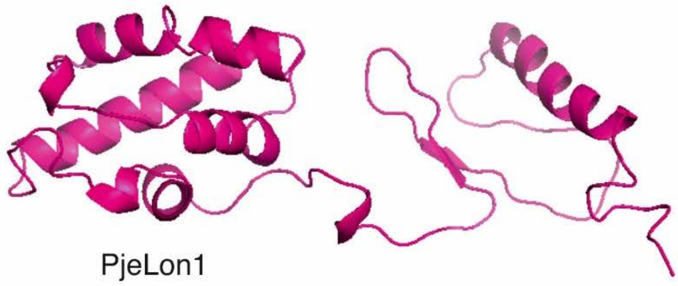

PjeLon1

***Pinus parviflora***

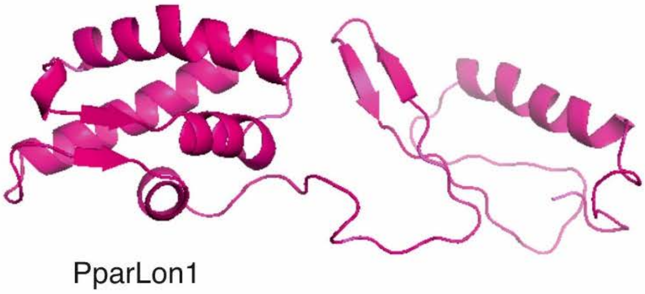

PparLon1

# Gymnosperms

***Pinus radiata***

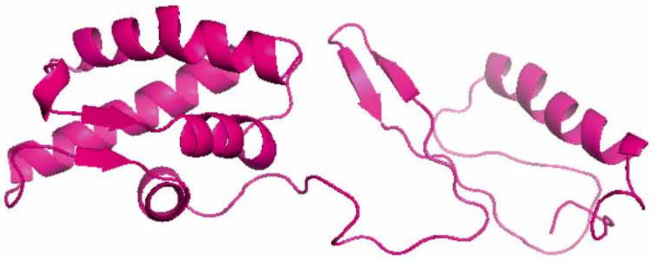

PraLon1

***Taxus baccata***

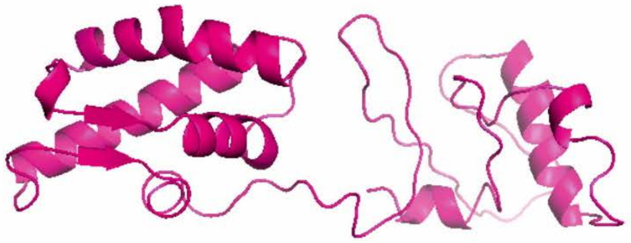

TbaLon1

# Angiosperms

***Amborella trichopoda***

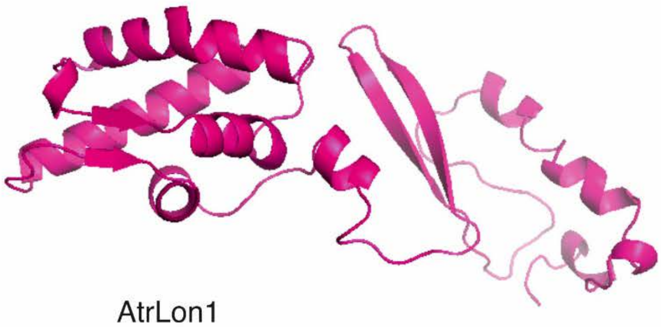

AtrLon1

# Angiosperms

## Monocots

***Ananas comosus***

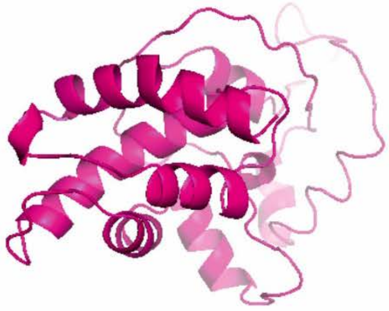

AcoLon1

***Brachypodium distachyon***

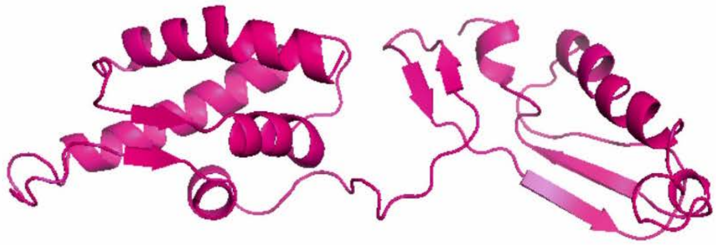

BdiLon1

***Brachypodium stacei***

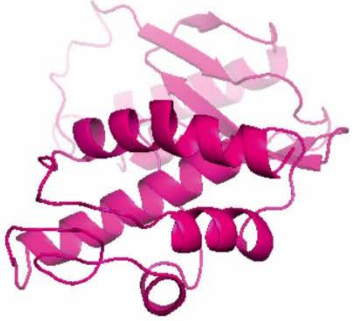

BstLon1

# Angiosperms

## Monocots

### *Oryza sativa*

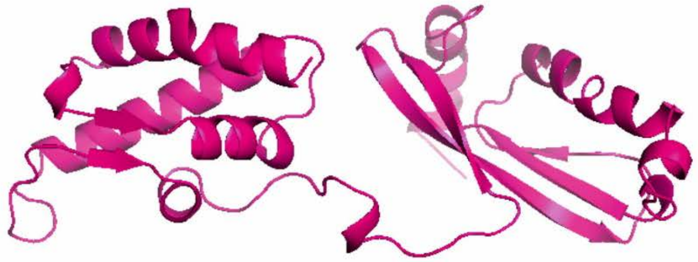

OsLonP1

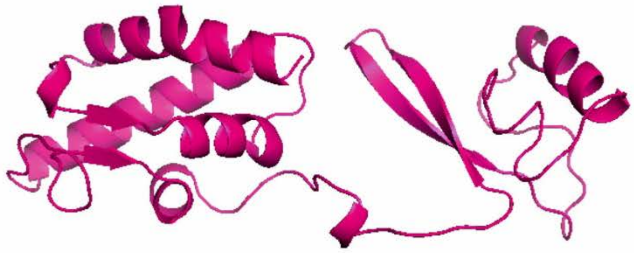

OsLonP3

### *Zostera marina*

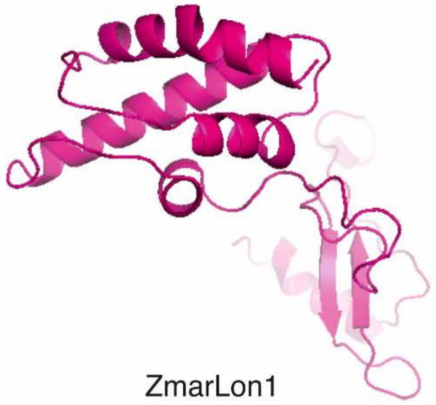

ZmarLon1

# Angiosperms
## Monocots

***Panicum hallii***

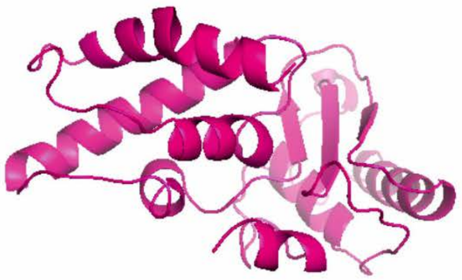

PhaLon1

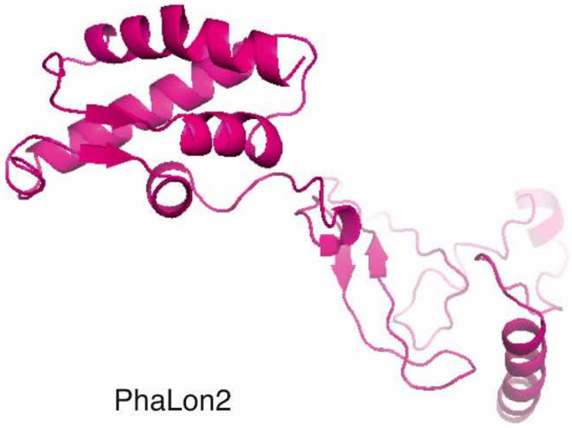

PhaLon2

# Angiosperms

## Monocots

***Sorghum bicolor***

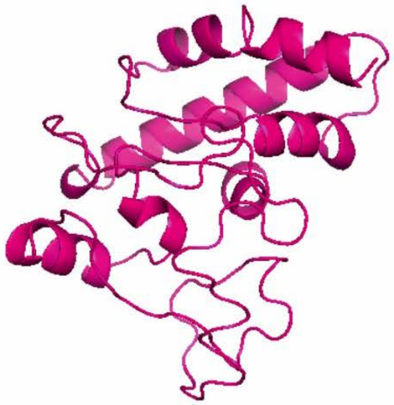

SbiLon1

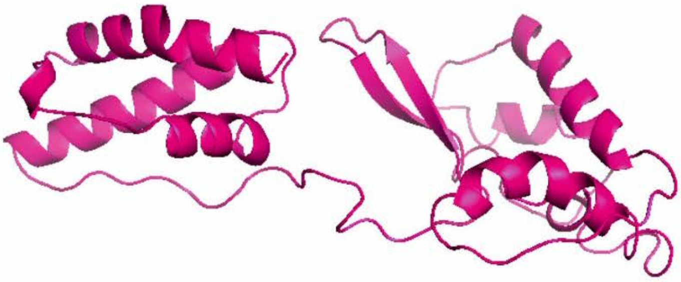

SbiLon2

# Angiosperms

## Monocots

***Setaria italica***

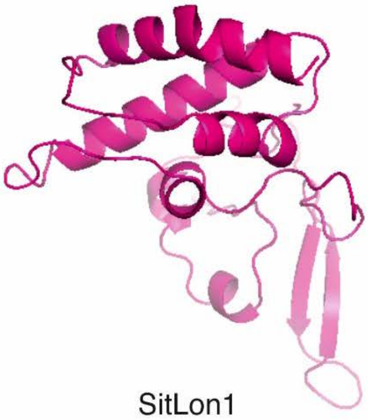

SitLon1

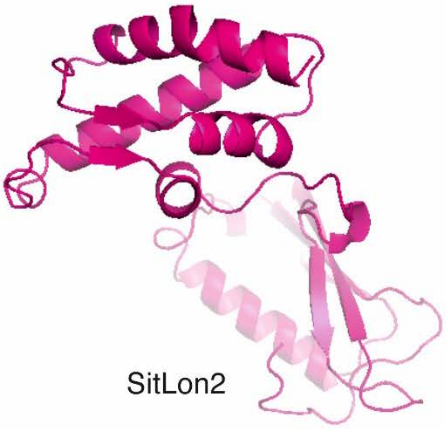

SitLon2

# Angiosperms

## Eudicots, Class: Brassicales

***Arabis alpina***

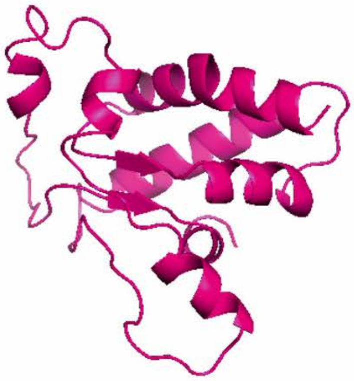

AaLon1

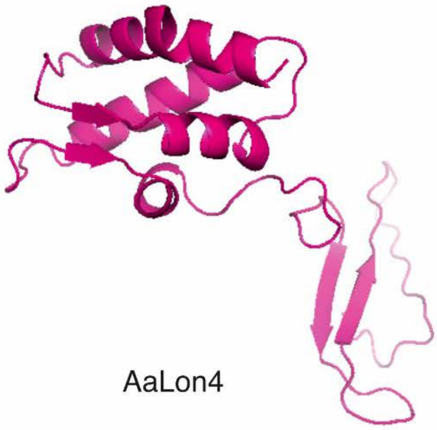

AaLon4

# Angiosperms

## Eudicots, Class: Brassicales

***Arabidopsis lyrata***

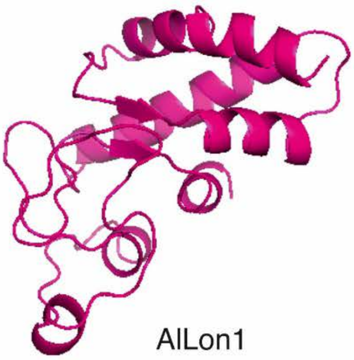

AlLon1

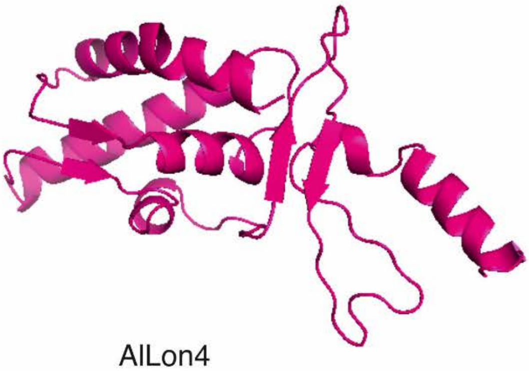

AlLon4

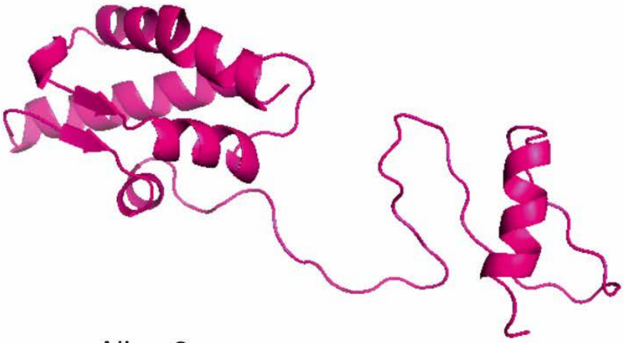

AlLon3

# Angiosperms

## Eudicots, Class: Brassicales

***Arabidopsis thaliana***

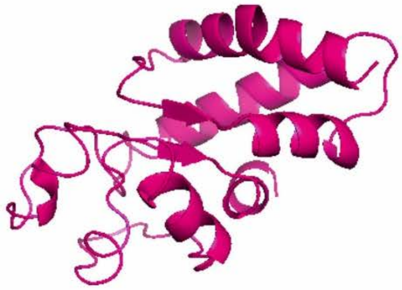

AtLon1

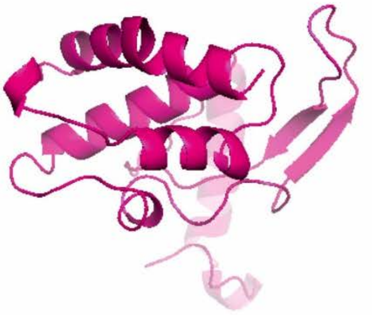

AtLon4

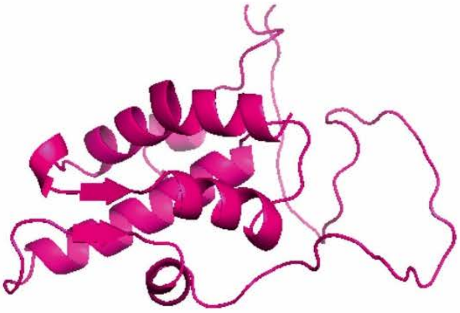

AtLon3

# Angiosperms

## Eudicots, Class: Brassicales

***Capsella grandiflora***

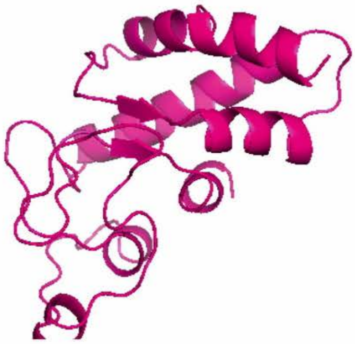

CgLon1

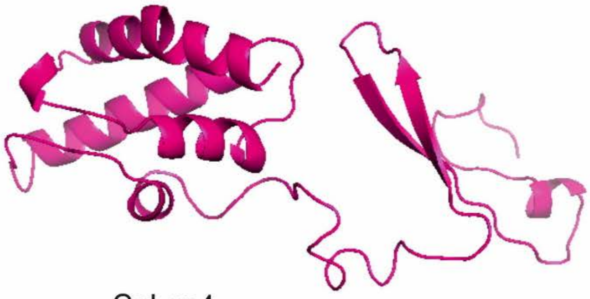

CgLon4

***Carica papaya***

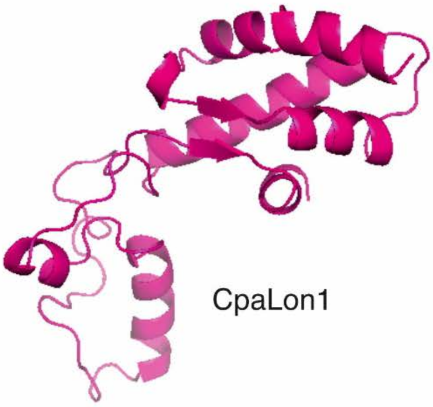

CpaLon1

# Angiosperms

## Eudicots, Class: Brassicales

***Capsella rubella***

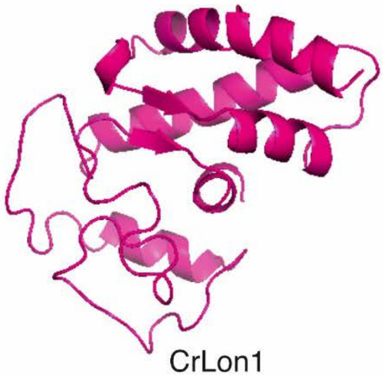

CrLon1

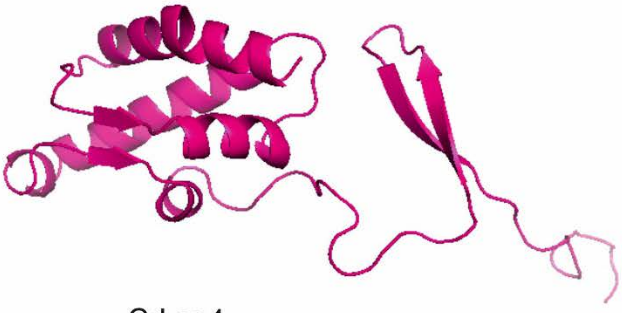

CrLon4

# Angiosperms

## Eudicots, Class: Sapindales

***Citrus clementina***

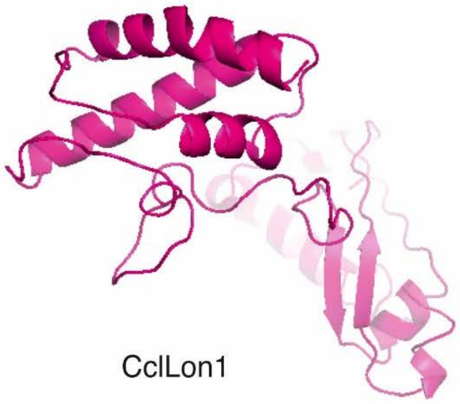

CclLon1

***Citrus sinensis***

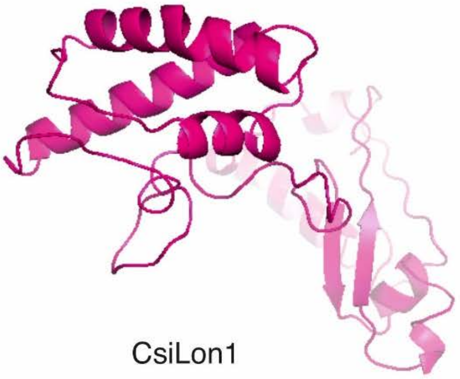

CsiLon1

# Angiosperms

## Eudicots, Class: Sapindales

***Anacardium occidentale***

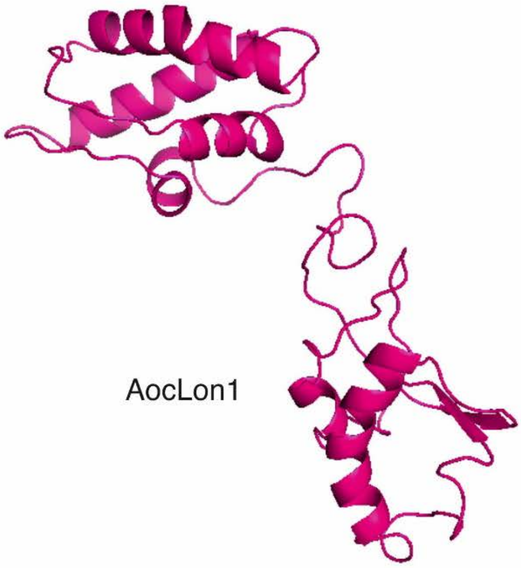

AocLon1

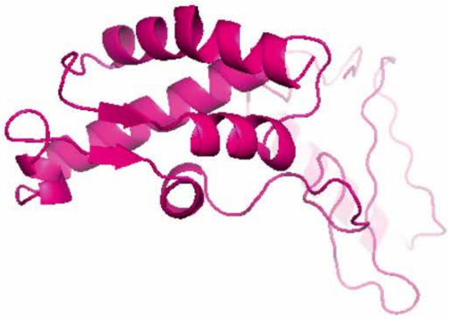

AocLon4

# Angiosperms

## Eudicots, Class: Rosales

***Prunus persica***

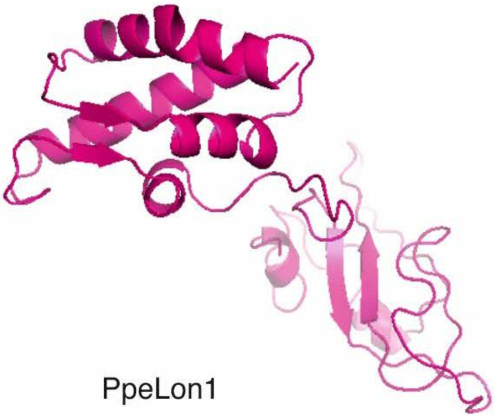

PpeLon1

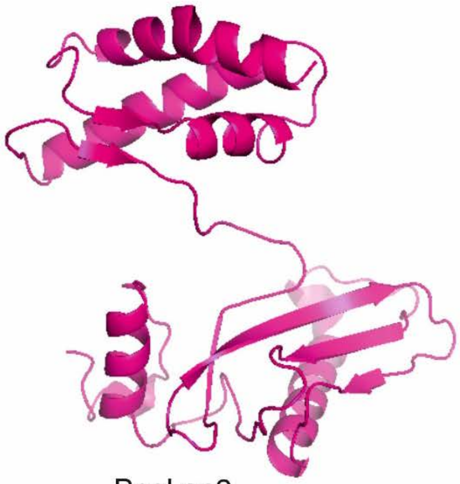

PpeLon2

# Angiosperms

## Eudicots, Class: Rosales

***Fragaria vesca***

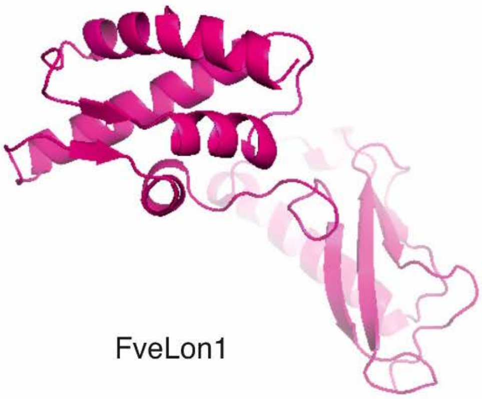

FveLon1

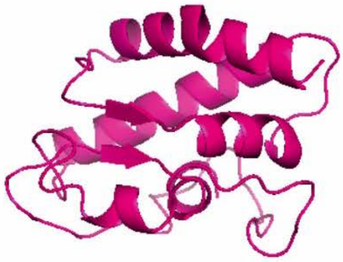

FveLon2

# Angiosperms

## Eudicots, Class: Fabales

***Glycine max***

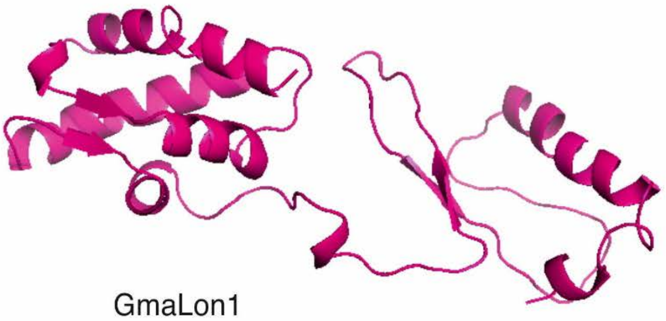

GmaLon1

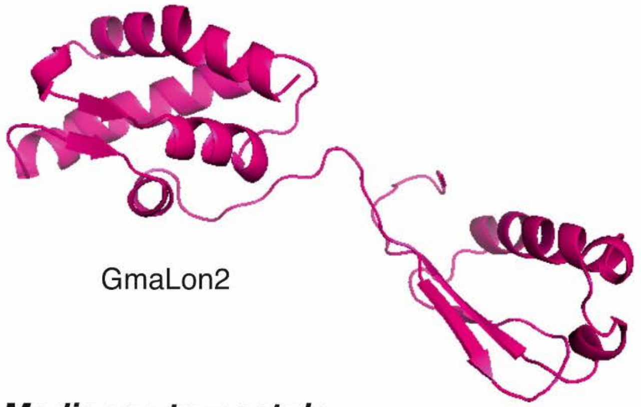

GmaLon2

***Medicago truncatula***

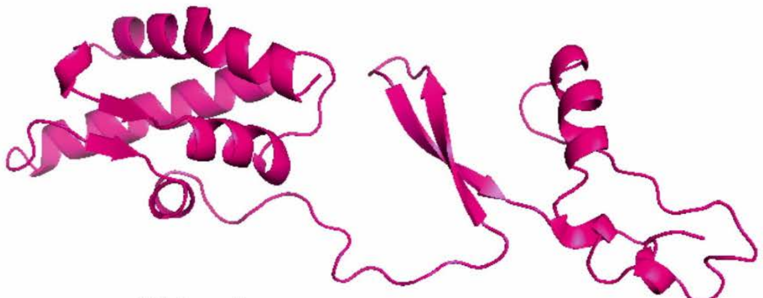

MtrLon1

# Angiosperms

## Eudicots, Class: Fabales

***Trifolium pratense***

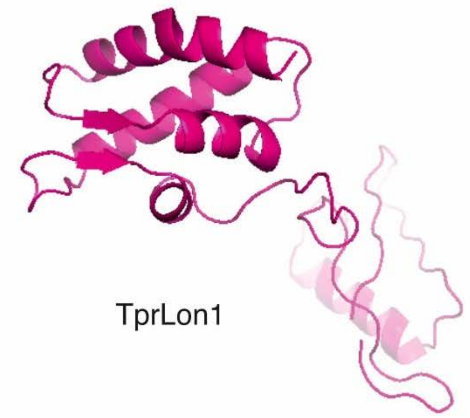


***Phaseolus vulgaris***

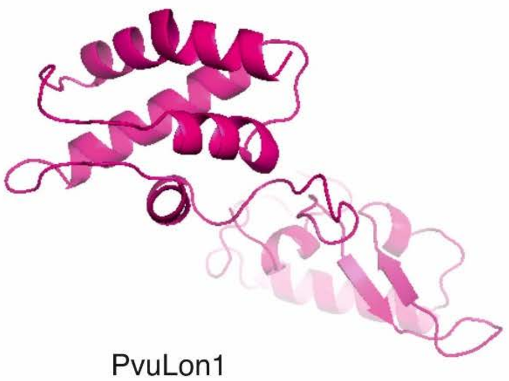

# Angiosperms

## Eudicots, Class: Malvales

***Gossypium hirsutum***

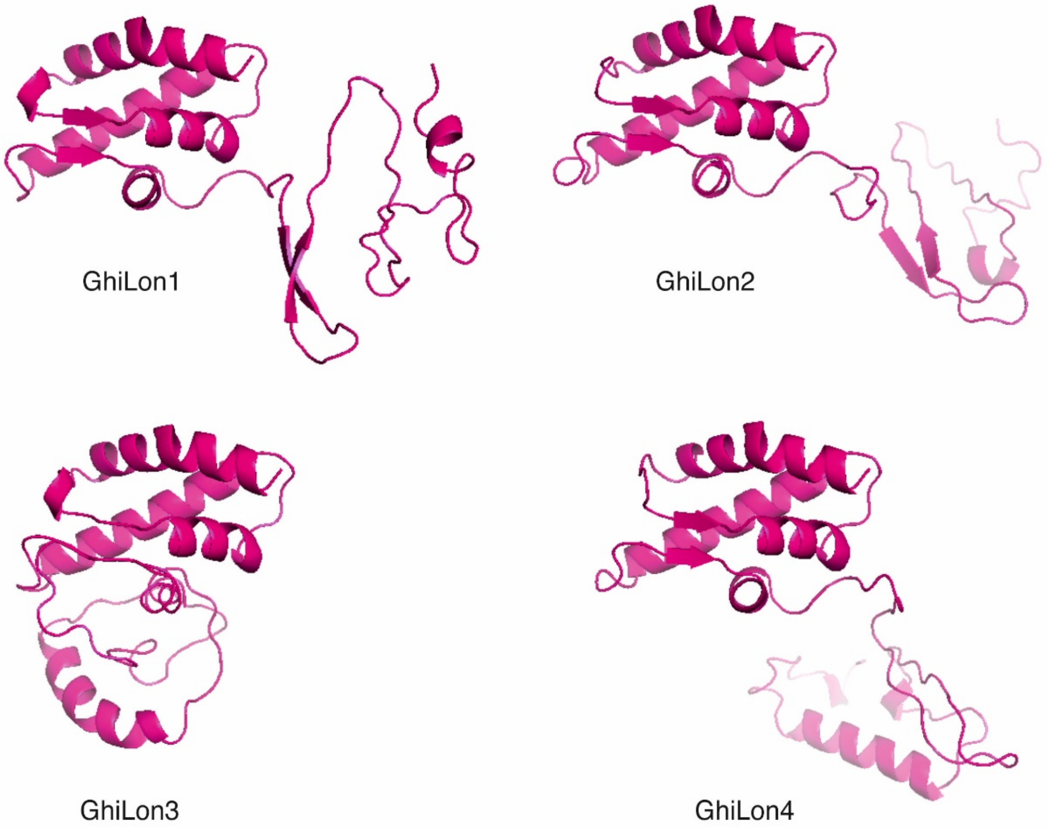

# Angiosperms

## Eudicots, Class: Malvales

### *Gossypium raimondii*

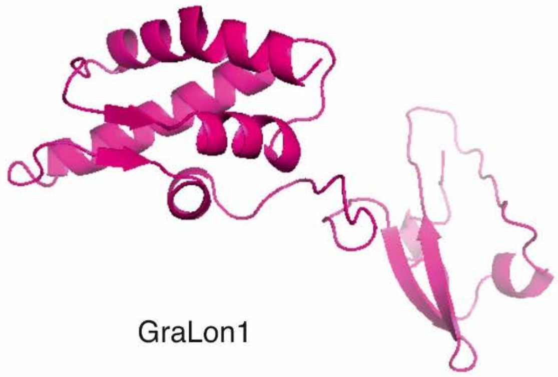

GraLon1

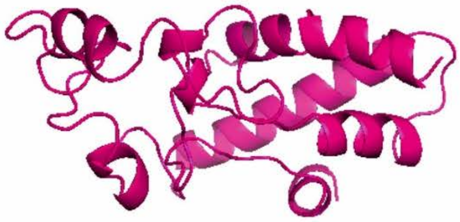

GraLon4

### *Theobroma cacao*

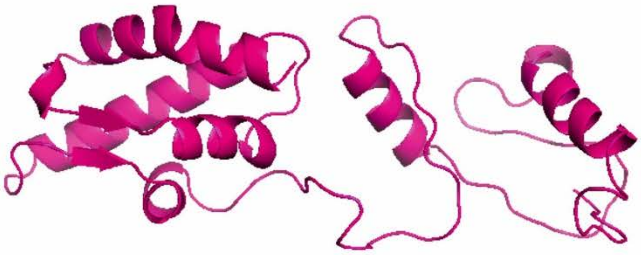

TcLon1

# Angiosperms

## Eudicots, Class: Malpighiales

### *Populus deltoides*

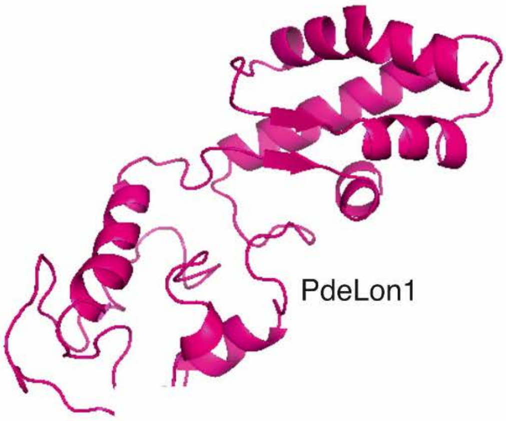

PdeLon1

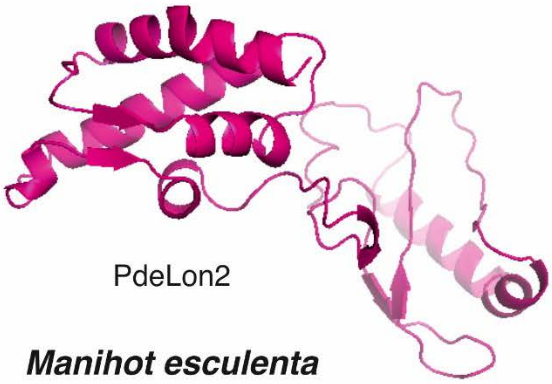

PdeLon2

### *Manihot esculenta*

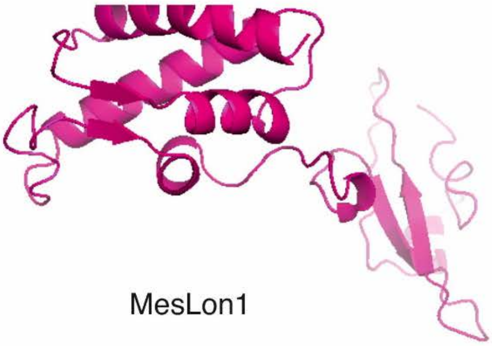

MesLon1

# Angiosperms

## Eudicots, Class: Malpighiales

***Populus trichocarpa***

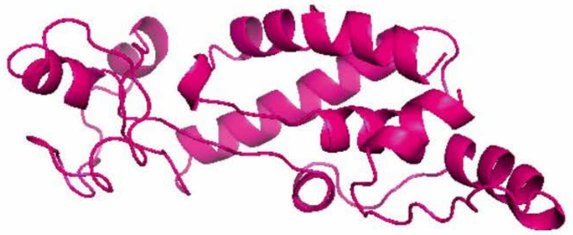

PtrLon1

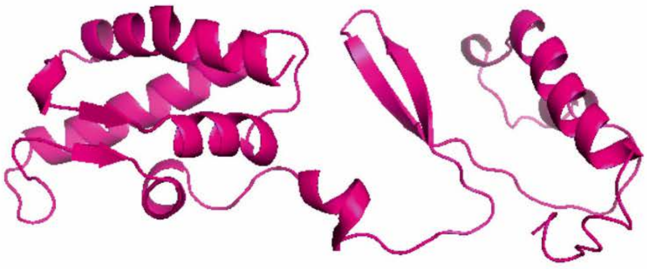

PtrLon2

***Salix purpurea***

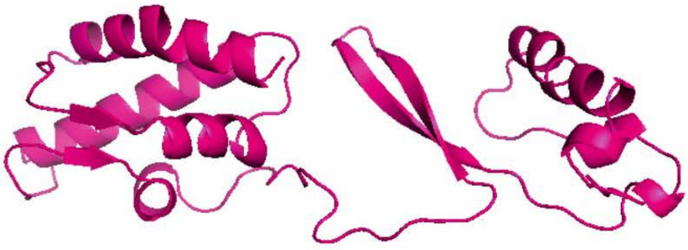

SpuLon1

# Angiosperms

## Eudicots, Class: Asterids

***Coffea arabica***

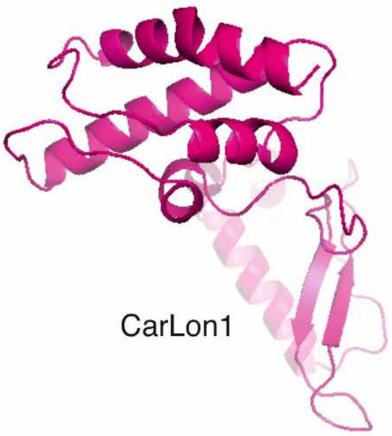


***Helianthus annuus***

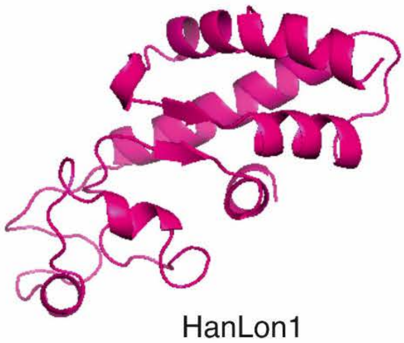


***Lactuca sativa***

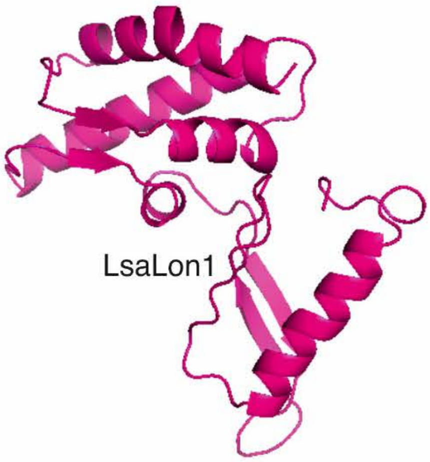

# Angiosperms

## Eudicots, Class: Asterids

***Mimulus guttatus***

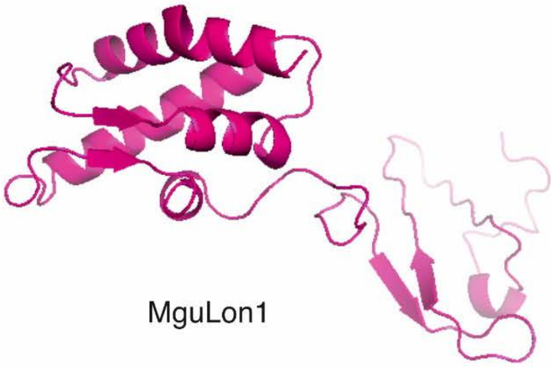

MguLon1

***Olea europaea***

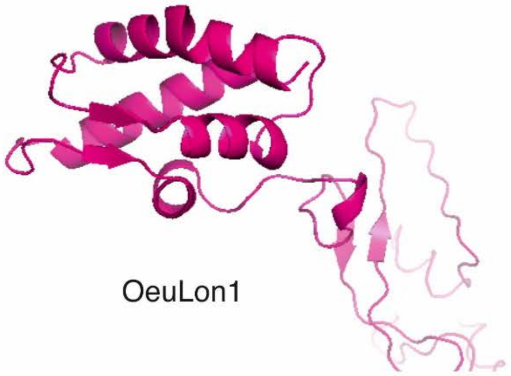

OeuLon1

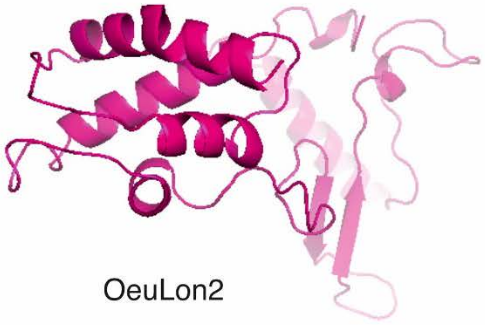

OeuLon2

# Angiosperms

Eudicots, Class: Myrtales

***Eucalyptus grandis***

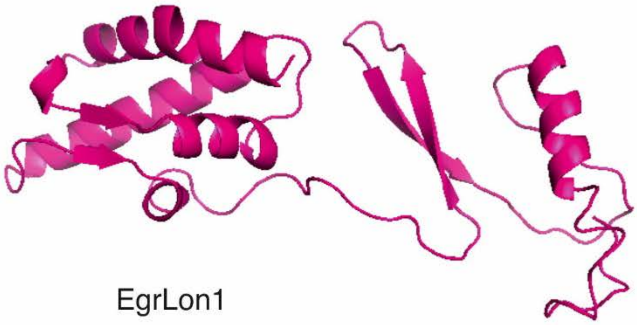

EgrLon1

# Angiosperms

Eudicots, Class: Cucurbitales

***Cucumis sativus***

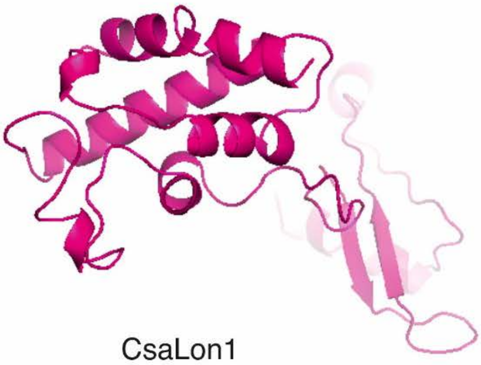

CsaLon1

# Animalia

***Homo sapiens***

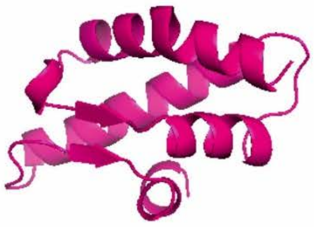

HsLonP1

***Pan troglodytes***

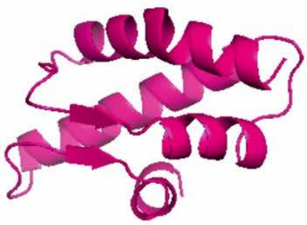

PtLonP1

***Bos taurus***

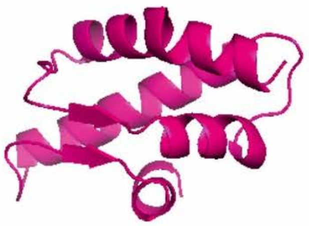

BtLonP1

***Canis lupus familiaris***

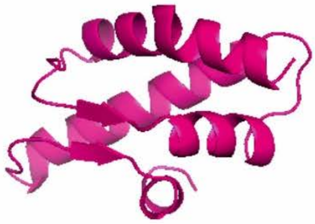

ClfLonP1

***Mus musculus***

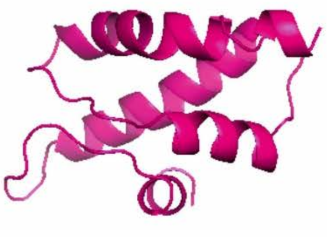

MmLonP1

***Rattus norvegicus***

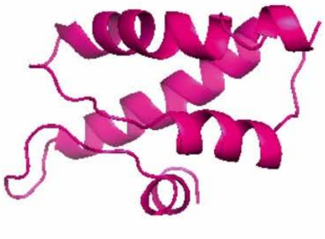

RnLonP1

# Animalia

***Cavia porcellus***

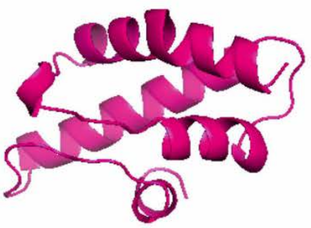

CpLonP1

***Salmo salar***

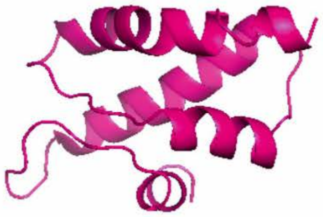

SsLonP1

***Callorhinchus milii***

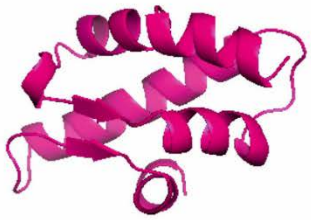

CmLonP1

***Danio rerio***

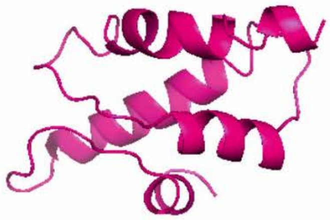

DrLonP1

***Meleagris gallopavo***

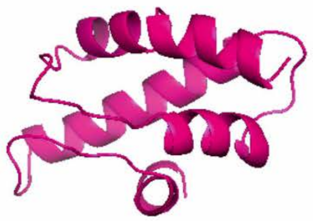

MgLonP1

***Ficedula albicollis***

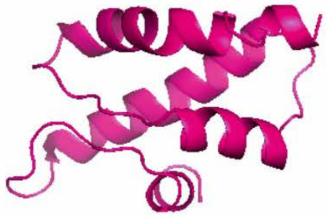

FaLonP1

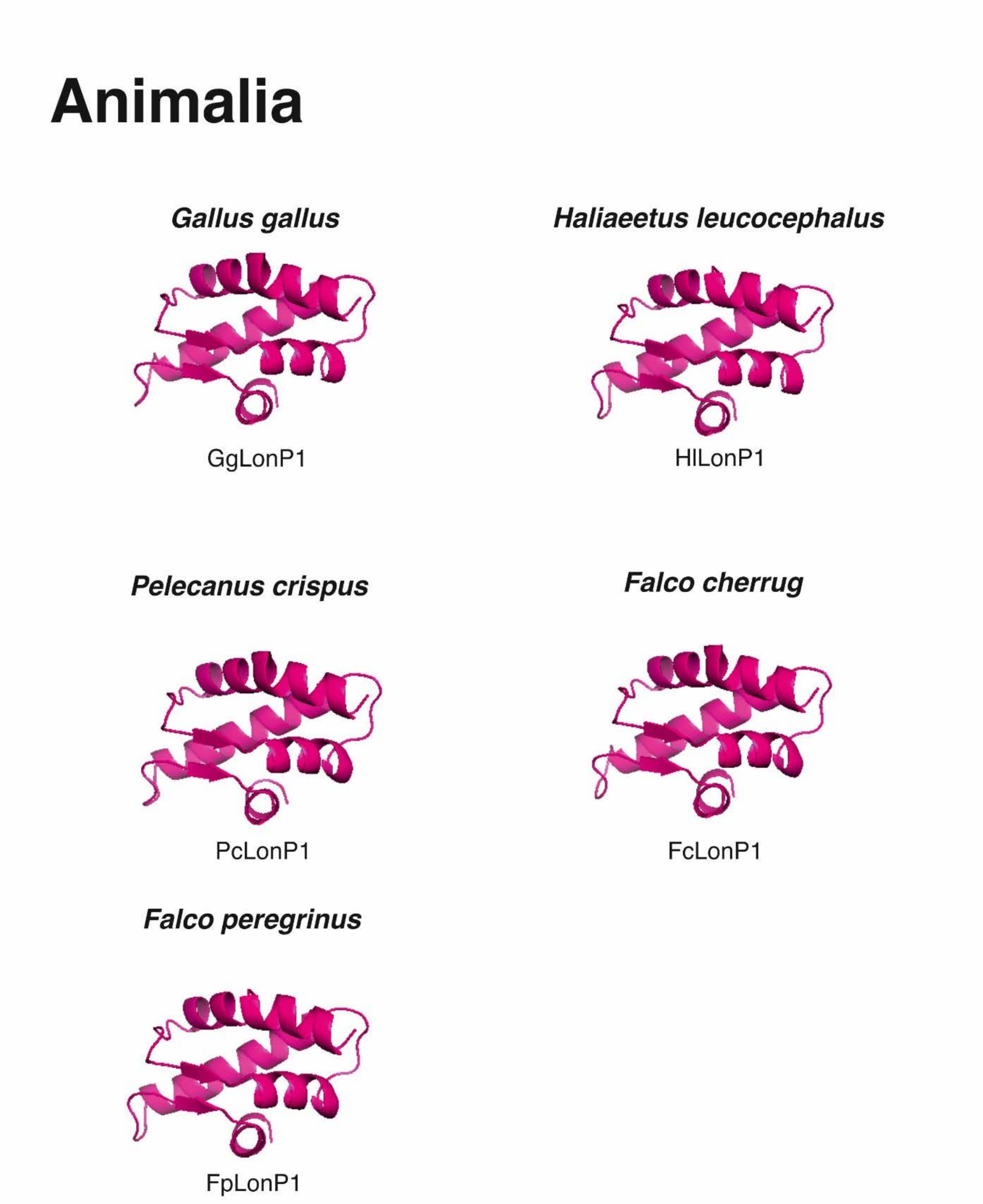


**Supplementary Fig. S6.** Molecular modeling of the sensor- and substrate-discrimination (SSD) domain reveals the functional features of Lon proteases. The Lon protein accessions and the coordinates of the SSD domains are shown in Supplementary Table S3. The analysis was performed on the basis of known crystallographic data mainly available from $AAA^+$ proteins and bacterial Lon proteases, which were automatically detected by the Phyre2 Protein Fold Recognition Server

(www.sbg.bio.ic.ac.uk/phyre2). The ribbon model was generated in PyMol (www.pymol.org).
